# *DWARF27* and *CAROTENOID CLEAVAGE DIOXYGENASE 7* genes regulate release, germination and growth of gemma in *Marchantia polymorpha*

**DOI:** 10.3389/fpls.2024.1358745

**Published:** 2024-06-25

**Authors:** Rubina Jibran, Jibran Tahir, Christelle M. Andre, Bart J. Janssen, Revel S. M. Drummond, Nick W. Albert, Yanfei Zhou, Kevin M. Davies, Kimberley C. Snowden

**Affiliations:** ^1^ Plant Development, The New Zealand Institute for Plant and Food Research Limited, Auckland, New Zealand; ^2^ Metabolite Traits in Plants, The New Zealand Institute for Plant and Food Research Limited, Palmerston, North, New Zealand

**Keywords:** *Marchantia polymorpha*, strigolactones, *DWARF27*, *Carotenoid Cleavage Dioxygenase 7*, gemma, gemma cup, ethylene

## Abstract

Strigolactones (SLs), a class of carotenoid-derived hormones, play a crucial role in flowering plants by regulating underground communication with symbiotic arbuscular mycorrhizal fungi (AM) and controlling shoot and root architecture. While the functions of core SL genes have been characterized in many plants, their roles in non-tracheophyte plants like liverworts require further investigation. In this study, we employed the model liverwort species *Marchantia polymorpha*, which lacks detectable SL production and orthologs of key SL biosynthetic genes, including *CAROTENOID CLEAVAGE DIOXYGENASE 8* (*CCD8*) and *MORE AXILLARY GROWTH 1* (*MAX1*). However, it retains some SL pathway components, including *DWARF27* (*D27*) and *CCD7*. To help elucidate the function of these remaining components in *M. polymorpha*, knockout mutants were generated for *MpD27–1*, *MpD27–2* and *MpCCD7*. Phenotypic comparisons of these mutants with the wild-type control revealed a novel role for these genes in regulating the release of gemmae from the gemma cup and the germination and growth of gemmae in the dark. *Mpd27–1*, *Mpd27–2*, and *Mpccd7* mutants showed lower transcript abundance of genes involved in photosynthesis, such as *EARLY LIGHT INDUCED* (*ELI*), and stress responses such as *LATE EMBRYOGENESIS ABUNDANT* (*LEA*) but exhibited higher transcript levels of *ETHYLENE RESPONSE FACTORS* (*ERFs*) and SL and carotenoid related genes, such as *TERPENE SYNTHASE* (*TS*), *CCD7* and *LECITHIN-RETINAL ACYL TRANSFERASE (LRAT)*. Furthermore, the mutants of *M. polymorpha* in the SL pathway exhibited increased contents of carotenoid. This unveils a previously unrecognized role for *MpD27–1, MpD27–2* and *MpCCD7* in controlling release, germination, and growth of gemmae in response to varying light conditions. These discoveries enhance our comprehension of the regulatory functions of SL biosynthesis genes in non-flowering plants.

## Introduction

Carotenoids are a group of isoprenoid metabolites produced by all photosynthetic organisms, including plants, algae, and cyanobacteria, which perform essential roles as accessory and photoprotective pigments (Sun et al., 2022). Carotenoids also play other roles in plants, for example, they provide precursors for strigolactone (SL) and abscisic acid (ABA) synthesis (Sun et al., 2022).

**Figure 1:**
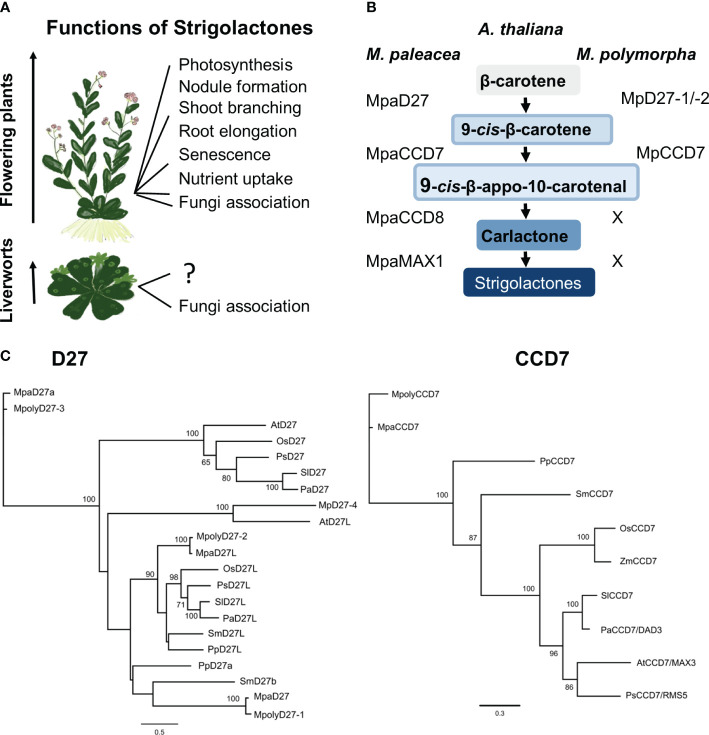
Strigolactones (SL) in flowering plants and liverworts. **(A)** SL functions in flowering plants and liverworts. **(B)** Homologs of SL precursor biosynthesis genes in *M. polymorpha* and *M. paleacea*. **(C)** Phylogenetic analysis of D27, D27L and CCD7 proteins across various plant species. Maximum likelihood phylogenetic trees were built by using PhyML with default settings (Guindon and Gascuel, 2003). Alignment was conducted using MUSCLE 3.8.425 (Edgar, 2004) within the Geneious Prime suite of software (https://www.geneious.com). Tree branch labels indicate probability percentages. The horizontal branch length is directly proportional to sequence divergence, as shown by the scale.

The evolution of mechanisms to communicate with other kingdoms of life has been central to plants adapting to the terrestrial environment (Brundrett, 2002; Bidartondo et al., 2011; Carella and Schornack, 2018; Aquino et al., 2021). One such adaptation to arise after the migration to land is believed to be the symbiotic interaction of plants with arbuscular mycorrhizal (AM) fungi to cope with nutrient starvation (de Saint Germain et al., 2013; Foo and Reid, 2013; Delaux et al., 2015). SLs play a key role in establishing interaction between plant roots and AM fungi (Yeum and Russell, 2002). Beyond their role as rhizosphere signaling molecule, SLs also regulate various aspects of plant growth and development, such as root and shoot architecture, and plant adaptation to stress responses as illustrated in **Figure 1A** (Yeum and Russell, 2002). In flowering plants, the SL pathway is highly conserved and well characterized, with key biosynthesis and signaling genes having been identified (Gomez-Roldan et al., 2008; Dun et al., 2013; Drummond et al., 2015, 2023). However, in primary land plant lineages such as bryophytes, the SL pathway requires further investigation (Walker et al., 2019).

Published data suggest that some plant lineages lack a full complement of biosynthetic genes necessary to produce SL (Walker et al., 2019). For example, hornworts lack *DWARF 27* (*D27*), *CAROTENOID CLEAVAGE DIOXYGENASE 7* (*CCD7*), and *MORE AXILLARY GROWTH 1* (*MAX1*) but possess *CCD8* and an ancestral form of *LATERAL BRANCHING OXIDOREDUCTASE* (*proto-LBO*), the functions of which are unclear. In the model liverwort species *M. polymorpha*, orthologs for *D27* and *CCD7* are present, but the key SL biosynthesis genes, *CCD8* and *MAX1*, and canonical SL metabolites have not been reported (Walker et al., 2019) (**Figure 1B**). In contrast, a close relative, *Marchantia paleacea*, possesses all the SL biosynthesis enzymes, exhibits growth suppression when treated with synthetic SLs, and interacts with AM fungi to enhance water and nutrient uptake, particularly phosphorous (Humphreys et al., 2010; Costa and Peralta, 2015; Sgroi and Paszkowski, 2020). This raises questions regarding the roles of D27 and CCD7 in *M. polymorpha*, and whether they contribute to plant adaptation responses.

Bryophytes, including the monophyletic lineages of hornworts, mosses, and liverworts, are thought to be diverged from the common ancestor they share with flowering plants roughly 400 million years ago (Shaw et al., 2011; Morris et al., 2018; Li et al., 2020). *M. polymorpha* (hereafter referred to as Marchantia) serves as a model plant for the liverworts because it is feasible to cultivate, has a small genome, and the genetic manipulations have been well established (Ishizaki et al., 2016; Poveda, 2020; Kohchi et al., 2021; Bowman et al., 2022). Marchantia produces distinct unisexual individuals, male and female, each with gametophyte and sporophyte generations. The dominant and haploid gametophyte form, contrasts with the dependent diploid sporophyte. Asexual propagation occurs through disc-like vegetative propagules known as gemmae. When conditions become conducive to sexual reproduction, such as long days and an abundance of far-red light, male and female gametophytes produce sex structures known as antheridiophores and archegoniophores, respectively. Liverworts lack true roots but possess root-like structures called rhizoids on the ventral side of the thallus. Rhizoids serve multiple functions, including nutrient and water absorption from soil, facilitating water distribution across the thallus, anchoring the thallus to a substrate, and forming mycorrhizal interactions.

To help establish the function of SL-related genes in Marchantia, we generated knockout mutants for the orthologs of *D27–1*, *D27–2* and *CCD7*. The mutant lines, along with wild-type plant, were quantitatively assessed for adaptive fitness when experiencing nutrient stress in either standard day/night or dark conditions. Our findings indicate that *MpD27–1, MpD27–2* and *MpCCD7* have actions in pathways controlling growth of gemmae. Gemmae are formed in the gemma cups, where they remain dormant until dislodged either by physical action, such as impact of rain, or the death of the parental plant. The *Mpd27–1, Mpd27–2* and *Mpccd7* mutants exhibit increased release of gemmae from the gemma cup and enhanced gemmae germination and growth in the dark compared with wild-type plant. Additionally, the mutants have increased transcript abundance for genes related to ethylene responses and carotenoid metabolism and reduced expression of genes linked to photosynthesis and stress responses. Furthermore, SL mutants in Marchantia exhibit increased amounts of carotenoids. Based on these results, we propose that *MpD27* and *MpCCD7* regulate the release of gemmae from the gemma cup and their subsequent germination and growth in response to varying light conditions.

## Methods

### Phylogenetic tree construction

Sequence alignments were performed on amino acid sequences deduced using MUSCLE 3.8.425 (Edgar, 2004) within the Geneious Prime suite of software (https://www.geneious.com). Candidate homologous sequences for Marchantia genes were obtained from Phytozome (https://phytozome-next.jgi.doe.gov/), Sol Genomics Network (https://solgenomics.net/) or NCBI (https://www.ncbi.nlm.nih.gov/), and are provided in **Supplementary Data S1-1**. Maximum likelihood phylogenetic trees were generated on the alignments using PhyML 2.2.4 (Guindon and Gascuel, 2003) with default settings.

### Plant lines and growth conditions

For plant transformation, *M. polymorpha* L. spores were obtained as described by (Albert et al., 2018). Plant lines were maintained asexually through the propagation of gemmae, either plated directly on 0.5× Gamborg’s B5 medium (Duchefa Biochemie, Haarlem, the Netherlands; 1% (w/v) sucrose, 1% (w/v) agar) or onto a sterile filter paper disc covering the medium. Standard culture conditions were 25°C, 16 h photoperiod and 30 µmol m^-2^ s^-1^ light intensity provided by cool white, fluorescent tubes. For carbon and nutrient deprivation experiments, gemmae (four independent transgenic lines and >50 biological replicates per treatment) were plated onto water agar medium solidified using 1% agar and grown for 10 days either in the light/dark or complete darkness.

### CRISPR/Cas9 mutagenesis

Three candidate Marchantia genes related to SL pathways were targeted: two genes for *DWARF 27*, *MpD27–1* (*Mp6g03970*) and *MpD27–2* (*Mp6g01750*), and a *CAROTENOID CLEAVAGE DIOXYGENASE 7* (*MpCCD7*/*Mp2g03280*). A gene encoding a protein known to regulate gemmae biology, SUPPRESSOR OF MORE AXILLARY GROWTH2-LIKE (*MpSMXL*/*Mp3g06310*), was also targeted. CRISPR/Cas9-based genome editing was performed as previously described (Albert et al., 2018). *Agrobacterium*-mediated transformation was used for transforming spores with a construct containing four guide RNAs per gene (guide sequences are provided in **Supplementary Data S1-2**). The four 20 bp guides/gene (N17VVR: protospacer adjacent motif) (Doench et al., 2014) were designed using Geneious Prime and synthesized by GenScript Biotech (NJ, USA) as a polycistronic sequence, consisting of 20 bp guide sequences fused to a modified sgRNA scaffold (Dang et al., 2015) separated by glycyl-tRNA sequences. The synthesized sequences were subcloned into pMpU6ENTR and recombined into the binary vector pMpGE010 (Kawamura et al., 2022) using LR Clonase II enzyme mix (Thermo Fisher Scientific). Following transformation, PCR amplification and DNA sequencing were used to find mutations within the target genes (**Supplementary Figure S1**, **Supplementary Data S1-3**). At least five independent mutant events were shown for each of the targeted genes. Mutants were found in the initial transgenics (T0) because the main life-stage of Marchantia is gametophytic and, thus, haploid. Mutant lines were propagated through gemmae (G1 generation) and re-sequenced to ensure establishment of non-chimeric lines, as gemmae are derived from single cells. Plants used for experiments were G2 generation or later. For most experiments, four independent lines were used, and all analysis were performed using the parental line as a control.

### Scoring the number of gemmae in a cup

To assay for variation in gemmae numbers, we assigned numbers to the gemma cups on the thallus of 8-week-old wild-type plant. The Marchantia thallus grows outwards from the meristem at the thallus branch tip, showing new gemma cups as it grows. Thus, the oldest cups are at the thallus base and the youngest next to the meristem. Gemma cups at the thallus base were called Gemma Cup 1 (GC1) and successive cups were numbered outwards to the tip of the thallus branch (GC7). For comparisons between transgenic lines and wild-type, GC3 and GC4 were used. To quantify gemmae numbers, gemma cups were removed from the thallus and counted using a magnifying lens.

### Scoring the gemmae growth and germination

To quantify gemmae growth during the standard day/night growing conditions, we photographed the growing gemmae and then measured gemmae area by using Image J (Schneider et al., 2012). The germination of gemmae held in the dark was scored by counting the number of elongated gemmae and gemmae that developed rhizoids.

### RNA sequencing analysis

The wild-type control and three transgenic lines *Mpd27–1#16*, *Mpd27–2#9* and *Mpccd7#17* were grown on half-strength Gamborg’s B5 medium for 5 weeks. For uniform tissue sampling, a 6 mm diameter disc encapsulating GC3 and GC4 was harvested, weighing approximately 160 mg per biological replicate (with three biological replicates per genotype). Total RNA was extracted using the Spectrum™ Plant Total RNA Kit (Sigma Aldrich) following the manufacturer’s instructions. RNA-sequencing library construction and sequencing was performed by Beijing Genomics Institute (Shenzhen, China) using DNBSEQ Eukaryotic Strand-specific mRNA library preparation and the DNBseq platform. The resultant reads were mapped onto *M. polymorpha* subsp. *ruderalis* genome version 6.1 (https://marchantia.info/) using HISAT2 (Kim et al., 2019) with default parameters. Read counts were then calculated by Feature Counts (Liao et al., 2014). Feature counts generated were further filtered for genes with lower counts and replicates where genes are not expressed at all using edgeR (filterByExp, with default settings, ensuring removal of genes with lower than 10 counts in all samples). This left a total number of 13,408 genes across all samples. The featurecounts were also processed for K means clustering (top most variable 2000 genes, 6 clusters) and Hierarchical clustering analysis using the iDEP application through the subtraction of each gene’s mean expression level (Ge et al., 2018).

The filtered featurecounts were then processed for pair-wise differential expression (DE) analysis using edgeR (QLFTest) (Robinson et al., 2010). Files for each comparison were then filtered for genes with FDR <0.05 and logFC +-1.5. These files are presented for each comparison and an Upsetplot was performed on all these treatments (https://upsetplot.readthedocs.io/en/stable/formats.html). Raw counts data are described in **Supplementary Data S1-4**, while differentially expressed genes (DEGs) are summarized in **Supplementary Data S1-5**.

### Ultra-high-performance liquid chromatography (hereafter referred to as UPLC) analysis of SL pathway mutants and wild-type plant

Ten milligrams of powdered freeze-dried thallus tissue from 8 weeks old Marchantia plants was mixed with 1 mL of acetone, homogenized using a vortex mixer for 30 s, and shaken at 4°C for 30 min to allow extraction of the lipophilic compounds. After centrifugation at 10,000 ×*g* for 10 min, the supernatant was collected. The extraction was repeated on the pellet using the same extraction solvent. Both supernatants were combined, evaporated until dry under a gentle stream of nitrogen, and resuspended in 1 mL of ethanol. The resulting extract was filtered through a 0.45 µm syringe filter and stored at −20°C prior to UPLC analysis. The quantification was carried out using a Waters Acquity UPLC system (Milford, MA, USA) equipped with a Photodiode Array Detector and a Mass Single-quadrupole Detector (QDa, Waters). An aliquot of 2 μL was injected onto an Acquity UPLC BEH C18 column (2.1 × 100 mm, 1.7 μm particle size, Waters) at 40°C, with a flow rate of 0.5 mL min^-1^. The solvents were: Solvent A: 0.1% formic acid in water, Solvent B: 0.1% formic acid in acetonitrile, and Solvent C: 0.1% formic acid in isopropanol (all v/v). The gradient elution program was: 0 min: 30% A, 66% B, 4% C, 0.5 min: 30% A, 66% B, 4% C, 10 min: 18% A, 72% B, 10% C, 13 min: 2% A, 78% B, 20% C, 17 min: 2% A, 78% B, 20% C, 17.1 min: 30% A, 66% B, 4% C, 20 min: 2% A, 78% B, 20% C. Carotenoids were detected at 420 nm and quantified as lutein or β-carotene equivalents using a six-point calibration curve and lutein and β-carotene calibration standards prepared from commercially available sources. Data acquisition with the QDa mass detector was undertaken in positive mode using the following conditions: total ion current (TIC) between *m/z* 100–1000 Da, capillary voltage: 1 kV, probe temperature: 600°C, and cone voltage: 5 V. Compounds were confirmed in selected ion recording (SIR) mode.

### Transcript quantification by droplet digital PCR

Droplet Digital PCR (ddPCR) was performed to validate the DEGs obtained from the RNA sequencing experiment. We used the BIO-RAD droplet digital PCR system to quantify transcripts of the target genes, with *MpACTIN* serving as the reference gene. The primer sequences are provided in **Supplementary Data S1-3**.

## Results

### Phylogenetic analysis of *MpD27–1*, *MpD27–2*, and *MpCCD7* to predict gene functions

Gene homologs for two steps of the SL biosynthetic pathway have been identified from Marchantia, *DWARF27–1* and *DWARF27–2*, (*MpD27–1/Mp6g03970 and MpD27–2/Mp6g01750*) and *CAROTENOID CLEAVAGE DIOXYGENASE 7* (*MpCCD7*/*Mp2g03280*). However, no gene sequences for two pivotal enzymes responsible for catalysing the final stages of this pathway, MORE AXILLARY GROWTH 1 (MAX1) and CAROTENOID CLEAVAGE DIOXYGENASE 8 (CCD8), have been identified in previous work (Delaux et al., 2012; Walker et al., 2019; Kodama et al., 2022) (**Figure 1B**). To examine the relationship of the *MpD27–1*, *MpD27–2* and *MpCCD7* candidate genes to those of other species, we conducted a phylogenetic analysis of D27 and CCD7 deduced amino acid sequences from representative species of liverworts, lycophytes, gymnosperms, and flowering plants (**Figure 1C**). Using iterative BLAST searches, a single CCD7 homolog was identified in Marchantia (*MpCCD7*), along with four homologous D27 genes (*MpD27–1*, -*2*, -*3* and -*4*). Of these, *MpD27–1* and *MpD27–2* were chosen for further analysis because they belong to the same clade. The phylogenetic analysis supported the identification of a single CCD7 ortholog and four closely related D27 homologs in Marchantia (**Figure 1C**).

Although the function of these genes in *M. paleacea* has not been characterized yet, it possesses a functional SL pathway (Kodama et al., 2022). Therefore, based on the close sequence identity, *MpD27–1*, *MpD27–2* and *MpCCD7* were hypothesized to perform functions related to SL metabolism and chosen for further functional analysis. Based on homology analysis these genes seem to regulate biosynthesis of the SL precursor, 9-cis-10′-apo-beta-carotenal. Considering that ancestors of liverworts had SLs (Kodama et al., 2022), we are referring to these genes in Marchantia as SL precursor biosynthesis genes, despite the absence of detectable SL production in Marchantia. This categorization is based on sequence similarity, probable evolutionary relatedness, and likely functional conservation.

### Generation of knockout mutant lines for *MpD27–1, MpD27–2* and *MpCCD7*

To investigate the biological functions of *MpD27–1*, *MpD27–2* and *MpCCD7*, CRISPR/Cas9 Marchantia knockout mutants were generated for each gene, using *Agrobacterium*-mediated transformation of spores. The resultant mutations ranged from single nucleotide insertions or deletions to larger modifications, with deletions extending up to 370 bp and insertions reaching 231 bp (**Supplementary Figure S1**). Most of the genetic changes were predicted to result in truncated proteins. Marchantia is dioicous, so spores for transformation must be generated from a cross of male and female lines, which can generate heterogeneous genetic background among sporelings (Ishizaki et al., 2016). Thus, to isolate the effects of the mutations from genetic background variability, multiple knockout lines were identified for each target gene. Four distinct mutant lines were consequently employed as biological replicates for all experiments (except transcriptomic analysis), in addition to the parental line. Additionally, based on initial analysis of the mutant lines suggesting a phenotypic change in gemmae biology and gemmae gemination on the mother plant, a CRISPR/Cas9 knockout mutant for *SUPPRESSOR OF MORE AXILLARY GROWTH2-LIKE* (*MpSMXL*/*Mp3g06310*) was generated, as this gene regulates karrikin signaling and gemmae numbers in Marchantia (Komatsu et al., 2023) and provided a useful comparative control.

### *MpD27–1, MpD27–2* and *MpCCD7* are required for regulation of gemma release from gemma cups

**Figure 2:**
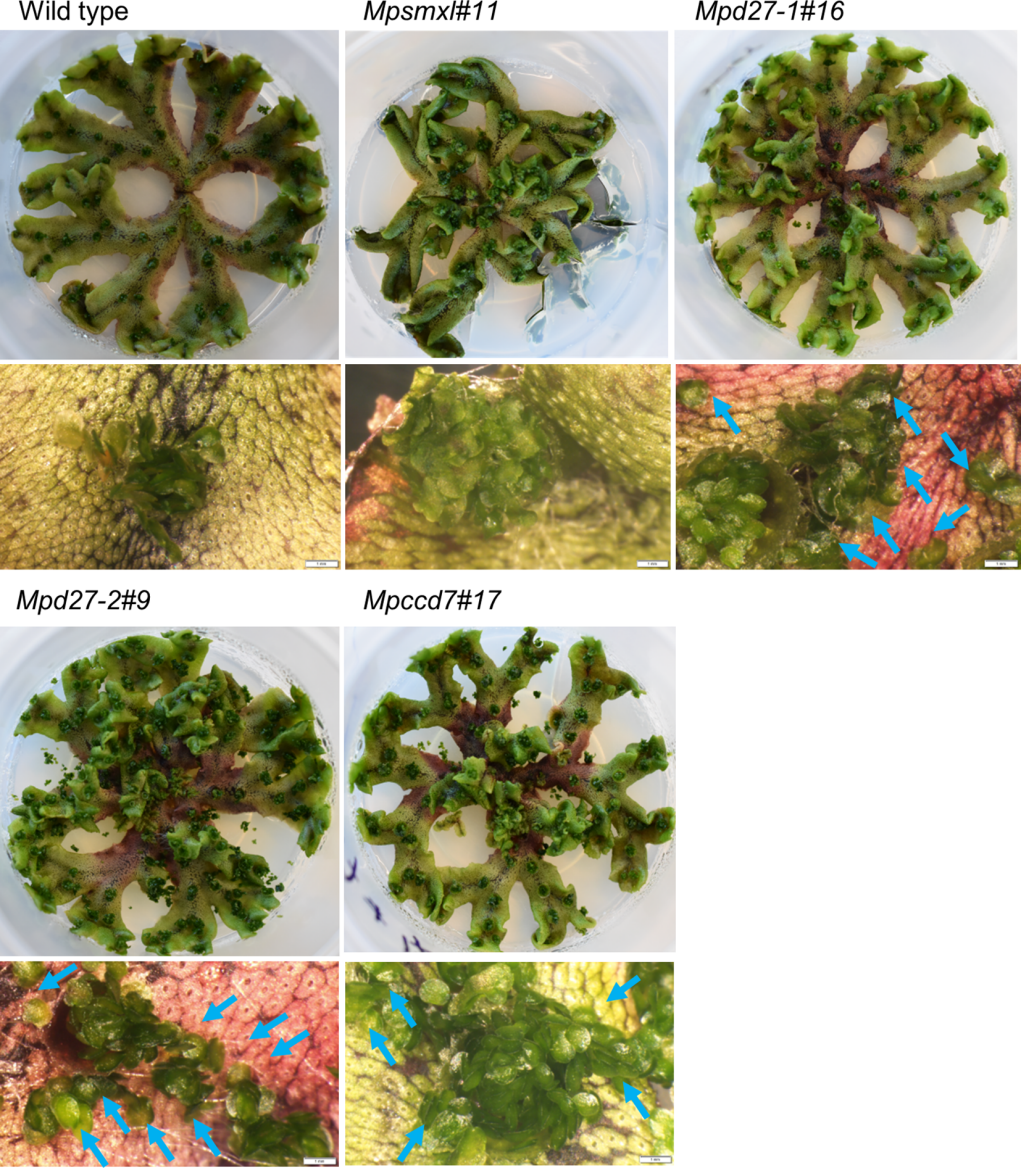
Phenotypes of wild-type and *Mpsmxl#11, Mpd27–1#16*, *Mpd27–2#9* and *Mpccd7#17* mutant plants. Plants were grown on complete media (1/2 strength Gamborg’s B5 medium supplemented with 1% sucrose and solidified with 1% agar) for 8 weeks at 25°C, 16-hour photoperiod and a light intensity of 30 mol m^-2^ s^-1^, provided by cool fluorescent tubes. Blue arrows indicate the gemmae released from the gemma cup. Scale bars indicate 0.5 cm for the entire plant and 1 mm for the thallus sections imaged with a light microscope.

**Figure 3:**
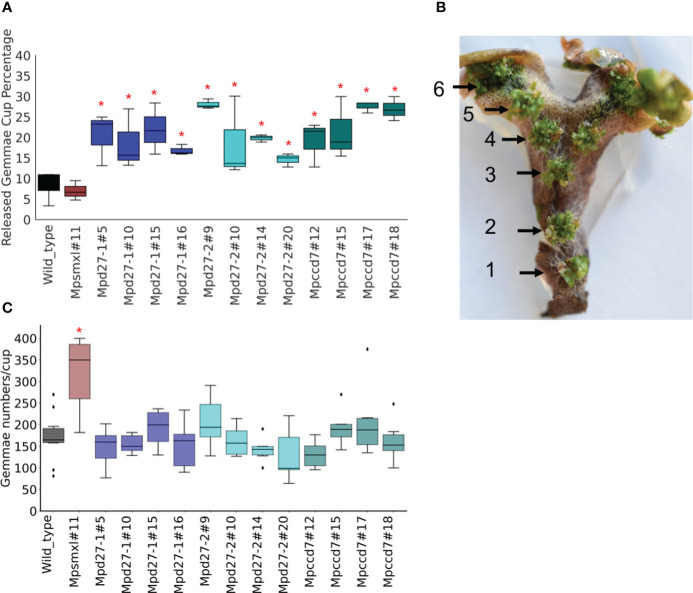
*MpD27–1, MpD27–2*, and *MpCCD7* regulate release of gemmae from gemmae cup. **(A)** Percentage of gemma cups **(GC)** was calculated by dividing the number of GC showing gemmae release with the total number of GC. **(B)** The wild-type thallus branch representing the numbering system used to study gemmae numbers in gemmae cups located at different positions. The numbers and black arrow heads indicate the positions of the gemma cups (GC) on the thallus, representing their location and relative age. Number 1 corresponds to the oldest GC, located closest to the thallus base, whereas number 7 refers to the youngest GC, situated nearest to the apical notch. **(C)** The combined gemmae numbers for GC3 and GC4 for the wild-type and mutant genotypes, including *Mpsmxl#11, Mpd27–*1#16, *Mpd27–2#9*, and *Mpccd7#17.* The statistical analysis was performed by using Student’s t-test in comparison with wild type and is represented as *p* < 0.05 (*). The data shown were collected from 8-week-old plants and represent six biological replicates.

In tissue culture conditions, the gemmae of wild-type plants typically remain within the gemma cups. However, *Mpd27–1*, *Mpd27–2*, and *Mpccd7* mutants displayed a higher percentage of gemma cups with gemmae outside of the cups compared with the wild-type control (**Figures 2**, **3A**). Gemmae, upon dispersal from the cup, initiate growth, a phenomenon also observable in SL mutants, as evidenced in **Supplementary Figure S2A**. Previously, it was reported that mutants of *MpSMXL* exhibited an increase in gemma number, retarded thallus growth and suppressed gemma dormancy in the dark (Mizuno et al., 2021; Komatsu et al., 2023). Given that karrikins are thought to potentially mimic the bioactivity of SLs, we hypothesized that an increase in the number of gemmae per cup could be a driving factor behind this gemmae escape phenomenon. The *Mpsmxl#11* mutant did not exhibit the visible increase in gemmae outside of the cup observed in the SL precursor mutants (**Figures 2**, **3A**). However, to appropriately test the hypothesis, we quantified the gemmae number for both the SL precursor mutants and wild-type. To facilitate this analysis, we first examined how the spatial position of the cups influenced gemmae numbers in wild-type genotype.

A numbering system was used based on the relative age of gemma cups. The oldest cup, situated at the thallus base, was designated as GC1 (Gemma Cup1) and younger cups closer to the apical notch were numbered consecutively from GC1 (reaching GC7), as illustrated in **Figure 3B**. No significant differences were observed in the number of gemmae within cups positioned at various locations along the thallus (**Supplementary Figure S2B**). For consistency, subsequent analyses concentrated on GC3 and GC4, with the gemmae from both cups combined for quantification. The *Mpd27–1*, *Mpd27–2*, and *Mpccd7* mutants had no significant difference in gemmae numbers from wild-type (**Figure 3C**). This suggested that the observed gemma cup release phenotype in the SL precursor mutants was not due to an increase in gemmae count. Thus, *MpD27–1*, *MpD27–2* and *MpCCD7* may have a role in suppressing release of gemmae from the gemma cup, thereby reinforcing gemmae dormancy while on the parent plant.

### *MpD27–1, MpD27–2* and *MpCCD7* regulate gemmae germination and growth in the dark

Given that *MpD27–1, MpD27–2* and *MpCCD7* influence gemmae dispersal, a process that typically initiates gemmae germination under favorable conditions, we investigated whether the genes also impact gemmae germination and growth. We compared gemmae growth under nutrient- and carbon-starved conditions either in the presence or absence of light. The mutant lines grown in the light did not show any significant differences in gemmae growth compared with wild type, as determined by comparative surface area (**Supplementary Figure S3**).

**Figure 4:**
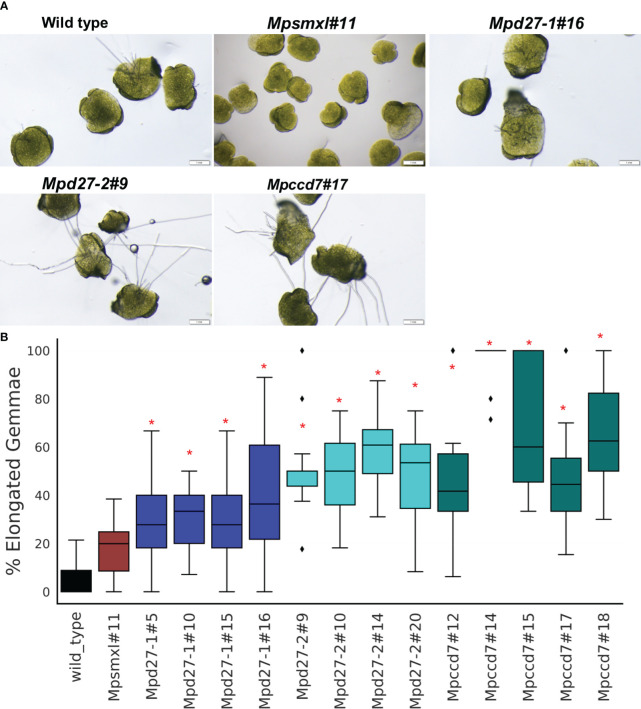
Dormant gemmae of wild-type, *Mpsmxl#11, Mpd27–1*, *Mpd27–2* and *Mpccd7* mutant plants grown on carbon- and nutrient-starved medium in darkness for 10 days. **(A)** Phenotypes of gemmae, with images taken on a light microscope. **(B)** Percentage of elongated gemmae. Scale bars represent 1 mm. Error bars denote mean ± SD (n = 50). Student’s t-test was employed to compare mutant lines with the wild-type genotype. Statistically significant differences against wild-type are represented as *p* < 0.05 (*).

The emergence of rhizoids is an indicator of the release of gemmae dormancy: gemma without rhizoids are considered dormant and those with visible rhizoids categorized as non-dormant (Eklund et al., 2015, 2018). Additionally, gemma germination and growth in the dark is typified by elongation of one side of the gemma (Mizuno et al., 2021). To evaluate germination of dark-grown gemmae, elongation and/or the presence of rhizoids were measured in mutants and the wild-type line. This assessment was impractical for light-grown gemmae, as all initially displayed gemmae growth and rhizoid emergence, until development was halted due to nutrient scarcity. Consequently, area-based metrics were exclusively used to gauge growth of light-grown gemmae. In dark conditions, all four lines of *Mpd27–1, Mpd27–2* and *Mpccd7* exhibited significantly higher numbers of gemmae with elongation compared with wild-type line (**Figure 4**). Additionally, the majority of the *Mpd27–2* mutant lines and all the *Mpccd7* mutant lines exhibited an increase in rhizoid production (**Supplementary Figure S4**). For *Mpsmxl#11* gemmae, growth appeared like wild-type line. The experimental evidence shows that *MpD27–1*, *MpD27–2* and *MpCCD27* are required to keep gemmae dormant under unfavorable (nutrient depleted and dark) conditions.

### Transcriptome analysis of *Mpd27–1*, *Mpd27–2* and *Mpccd7* mutants

**Figure 5:**
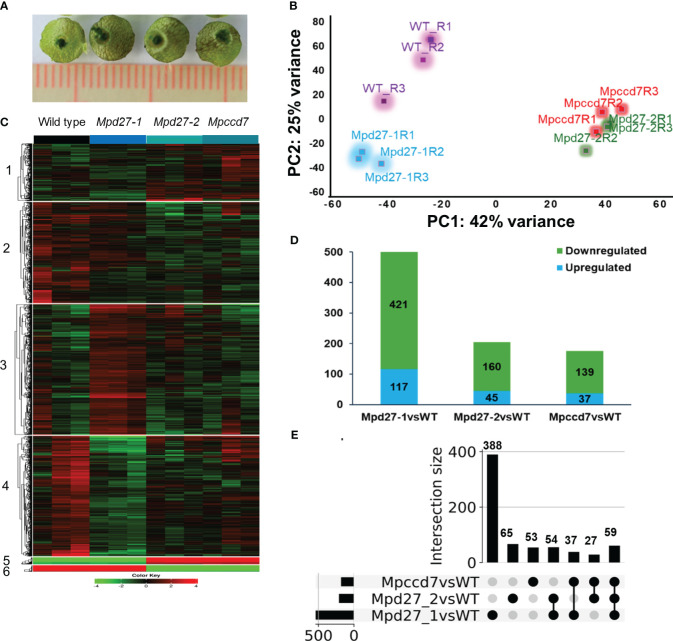
Transcriptome analysis of wild-type and *Mpd27–1*, *Mpd27–2* and *Mpccd7* genotypes. **(A)** Representative samples of the thallus tissue used for RNA sequencing analysis. **(B)** Principal Component Analysis (PCA) plot displaying variability within the expression dataset. **(C)** A heatmap of k means clustering applied on normalized (Nr) data through gene standardization. **(D)** DEGs in *Mpd27–1#16*, *Mpd27–2#9* and *Mpccd7#17* mutants compared with wild-type genotype. The *p*-values were calculated using Fisher’s exact tests. Significance analysis for clustering was conducted on normalized count data via ANOVA, with a false discovery rate (FDR) of < 0.05. **(E)** UpSet plot of differentially expressed genes from RNA sequencing at a log2 fold change (log2FC) of ±1.5 and FDR at <0.05. The black circle represents the set membership.

To find changes in mRNA abundance associated with gemma release, germination, and growth, comparative transcriptome analysis of *Mpd27–1*, *Mpd27–2* and *Mpccd7* mutants and wild-type control was conducted, focusing on evaluating the thallus tissue surrounding GC3 and GC4 (**Figure 5A**). Principal Component Analysis (PCA) demonstrated that biological replicates within each genotype were grouped closely (**Figure 5B**), whereas distinct groupings were observed among different genotypes, affirming the reliability of the data and the distinctiveness of phenotype between mutant and wild type. The raw counts and results of DEG analysis are given in **Supplementary Data S1-4, S1-5**.

Hierarchical clustering of the transcriptomic data grouped the top 2,000 most variable genes into two main clusters (**Supplementary Figure S5**, **Supplementary Data S1-6**). Cluster 1 included the transcript data from *Mpccd7* and *Mpd27–2*, while cluster 2 was composed of wild-type and *Mpd27–1.* This indicated that *Mpd27–2* and *Mpccd7* exhibited similar gene expression patterns, whereas *Mpd27–1* differed from other genotypes. Using K-means clustering yielded the expression patterns into notable six clusters (**Figure 5C**, **Supplementary Data S1-7**). Notably, clusters 4 and 2 exhibited the most contrasting profiles based on genotype-based differences. Furthermore, cluster 5 and 6 consisted of genes associated with the genotype’s sex.

To identify the genes that were differentially expressed in a pair-wise fashion between SL mutants and wild type we kept genes that showed log2FC of ± 1.5 at FDR <0.05. *Mpd27–1* had 421 downregulated and 117 upregulated genes, *Mpd27–2* had 160 downregulated and 45 upregulated genes, and *MpCCD7* showed 139 downregulated and 37 upregulated genes (**Figure 5D**, **Supplementary Data S1-7-S1-9**).

Employing UpSet plot on this dataset (**Figure 5E**, **Supplementary Data S1-8**), identified that 59 genes were differentially regulated across all three mutants. Specifically, 27 genes showed differential expression between *Mpccd7* and *Mpd27–2*, while 37 genes differed between *Mpd27–1* and *Mpccd7*. Among the detected DEGs, with the FDR/fold-change criteria used of <0.05/>1.5-fold, 388 out of 538 were associated only with *Mpd27–1*. In contrast, far fewer DEGs were found only in the *Mpd27–2* and *Mpccd7* datasets with these criteria, being 65 out of 205 and 53 out of 176, respectively.

### Transcriptomic analysis reveals molecular mechanisms of release, germination, and growth of gemmae

**Figure 6:**
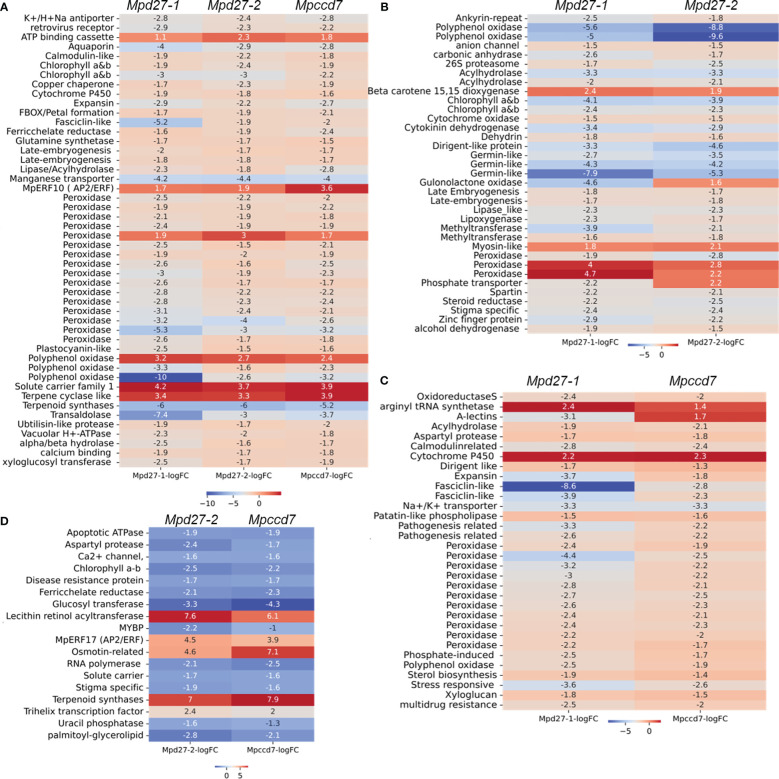
DEG comparisons among *Mpd27–1#16vsWT*, *Mpd27–2#9vsWT*, and *Mpccd7#17vsWT*. The heatmaps illustrate the log2 FC of genes in comparison to wild-type control highlighting genes common across **(A)** *Mpd27–1#16*, *Mpd27–2#9* and *Mpccd7#17*, **(B)** *Mpd27–1#16* and *Mpd27–2#9*, **(C)** *Mpd27–1#16* and *Mpccd7#17*, and **(D)** *Mpd27–2#9* and *Mpccd7#17*.

To enhance the clarity of our findings, we concentrated on analyzing DEGs that exhibited consistent changes across all three mutants. Specifically, we focused on genes predicted/known to have biological functions crucial to essential plant developmental processes including photosynthesis, stress adaptation, and terpenoid metabolism (**Figure 6**, **Supplementary Data S1-9**). Notably, genes encoding early light induced (ELI) proteins (including *Mp4g18580* and *Mp4g18590*), peroxidases (e.g., *Mp5g01640* and *Mp3g07970*), an α/β hydrolase (including *Mp2g15700*), and late embryogenesis abundant (LEA) proteins (including *Mp4g14840* and *Mp4g14870*) were predominantly downregulated. Conversely, *Mp7g00860*/*MpERF10*, an ethylene response factor, demonstrated increased transcript levels. Genes associated with terpenoid metabolism exhibited variable responses. For example, *Mp3g13150*/*MpTPS6* was significantly upregulated with a log2FC of 3.3, whereas *Mp3g21760*/*MpTPSL18* was downregulated with a log2FC of -5.9 (**Figure 6A**, **Supplementary Data S1-9**). Interestingly, the transcript abundance of *Mp4g19090*/*MpTPSL23* was higher in *Mpd27–2* and *Mpccd7* mutants compared to the *Mpd27–1* mutant (**Supplementary Data S1-9**).

The DEGs associated with photosynthesis, hormones, and stress adaptation were shared between *Mpd27–1* and *Mpd27–2* mutant RNAseq datasets (**Figure 6B**, **Supplementary Data S1-9**). For example, genes including *CAB*/*ELI* (e.g., *Mp4g18610* and *Mp4g18620*), *GERMIN-LIKE* (e.g., *Mp5g00880*, *Mp5g00920*, and *Mp5g13870*), and ABA-responsive e.g., *DEHYDRIN* (*Mp6g15610*) and *LEA* (e.g., *Mp7g06630* and *Mp4g14880*) were downregulated. At the same time *BETA-CAROTENE DIOXYGENASE* (*Mp4g01260*) was upregulated in both mutants. Similarly, DEGs between *Mpd27–1* and *Mpccd7* were mostly associated with stress responses. For example, *Mp4g02840* (induced by phosphate), *Mp8g18620*, (A-type lectin), and *Mp5g05460* (known as fasciclin-like) were downregulated, whereas *Mp6g18420* (a cytochrome P450), showed an increase in expression (**Figure 6C**, **Supplementary Data S1-9**). The downregulated genes common between *Mpd27–2* and *Mpccd7* included *CAB*/*ELI* (e.g., *Mp4g18560*), Stigma specific (e.g., *Mp5g11400*) and *PALMITOYL GLYCEROLIPID* (e.g., *Mp6g04480*) (**Figure 6D**, **Supplementary Data S1-9**). Conversely, genes such as *MpERF17*/*Mp4g22280*, *TRIHELIX16*/*Mp3g10500*), *MpTPSL23*/*Mp4g19090* and *LRAT*/*Mp3g10470* were upregulated. Taken together, these findings indicate that SL precursor mutants affected many genes regulating essential biological functions such as photosynthesis, ethylene signaling, stress responses, terpenoid and carotenoid metabolism.

**Figure 7:**
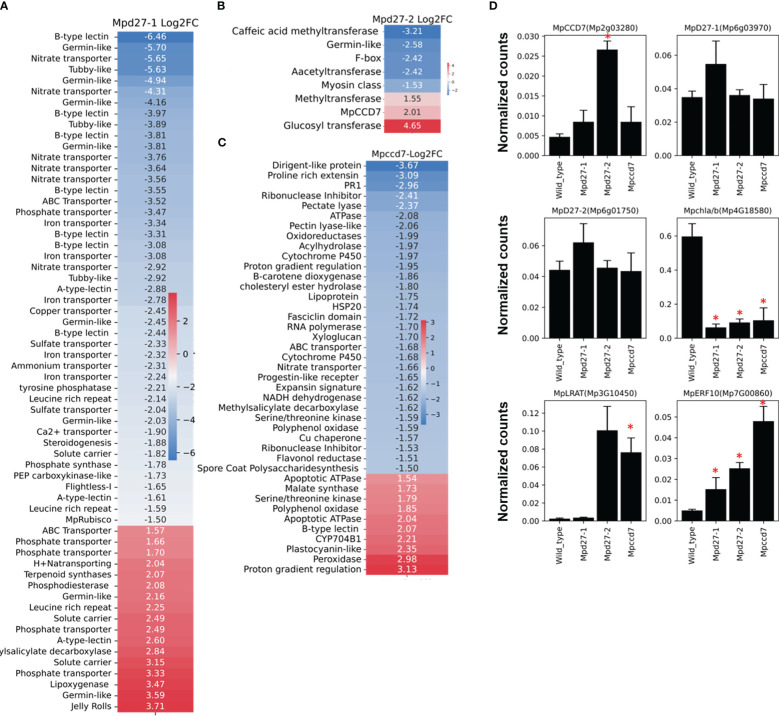
Heatmaps of the log2 FC of genes unique to *Mpd27–1#16* **(A)**, *Mpd27–2#9* **(B)**, and *Mpccd7#17* **(C)** mutants compared with the wild-type control. **(D)**. Digital Droplet PCR (ddPCR) analysis. The data represent three biological replicates. The y-axis represents the normalized counts, and the x-axis displays the gene names. The expression data were normalized against *MpACTIN*. The statistical analysis was performed by using Student’s t-test in comparison with wild type and is represented as *p* < 0.05 (*).

The transcriptome profile unique to *Mpd27–1* included many genes linked to stress response and cellular transport (**Figure 7A**, **Supplementary Data S1-9**). The ten lectin-related genes, involved in defense and communication responses against biotic and abiotic stress, were suppressed, exhibiting log2FC from -1.6 to -6.4. For example, A-type lectins *Mp1g18730* with a log2FC of -2.59 and B-type lectins *Mp8g18670* with a log2FC of -6.46 were downregulated. Similarly, transport-related genes involved in transport of iron (including Mp*2g25340* with a log2FC of -3.3), phosphate (including *Mp4g16570* with a log2FC of -3.46), and nitrate (including *Mp4g03070* with a log2FC of -5.6) showed decreased transcript abundance. In contrast, some phosphate transporters like *Mp4g11070* were upregulated with a log2FC of 3.3. Furthermore, terpenoid synthase (*Mp6g04630*/*MpMTPSL10*) increased by a log2FC of 2, and LOX2 (*Mp2g12180*) was upregulated with a log2FC of 3.46.

In *Mpd27–2*, *MpCCD7* (*Mp2g03280*) was upregulated 4-fold, suggesting that the mutant might be responding to a defect in a metabolite biosynthesis process regulated by *MpD27–2* (**Figure 7B**, **Supplementary Data S1-9**). This change could represent a compensatory mechanism for reduced flux within the pathway or be a result of feedback regulation. These findings suggest a potential link between *MpD27–2* and *MpCCD7* in the same metabolic pathway.

The *Mpccd7* mutant showed 3.6-fold increase in transcript abundance of *9-CIS-EPOXYCAROTENOID DIOXYGENASE* (*Mp2g07800/MpNCED*) (**Figure 7C**, **Supplementary Data S1-9**). Other genes with notable reduced expression included *SPORE COAT POLYSACCHARIDE* (*Mp3g15460*) with a log2FC of -1.5 and *FASCICLIN-LIKE ARABINOGALACTAN* (*Mp5g05390*) with a log2FC of -1.7. In contrast, *MALATE SYNTHASE* (*Mp8g16290*), *MpCYP704-like8* (*Mp3g10820*), and *MpNBS-LRR5* (*Mp3g09150*) were upregulated.

### Digital droplet PCR confirms the differential gene expression patterns found using RNAseq

Digital Droplet PCR (ddPCR) was used to validate the RNA-Sequencing findings, by examining six genes identified as altered in the mutant lines (**Figure 7D**). The ddPCR results confirmed the differential expression of the genes compared with the wild-type control. For example, *MpCCD7* (*Mp2g03280*) expression was increased >2-fold in *Mpd27–2*, while *MpERF10 (Mp7G00860)* exhibited a more than 4-fold increase in *Mpd27–2* and an over 8-fold increase in *Mpccd7*. In contrast *MpCAB* (*Mp4G18580*) was -5-fold downregulated in the *Mpd27–1*, -3.6-fold in *Mpd27–2* and -2.45-fold in *Mpccd7* mutants. These results are consistent with the RNAseq data, thereby validating the observed differential gene expression.

### The SL mutants showed altered accumulation of carotenoid compounds

**Figure 8:**
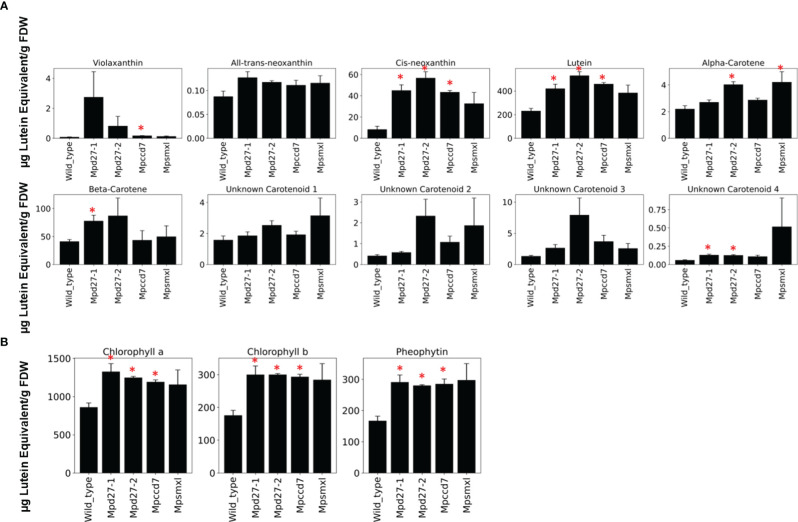
A targeted UHPLC analysis of *Mpd27–1#16*, *Mpd27–2#9*, *Mpccd7#17*, *Mpsmxl#11* and wild-type. **(A)** The amounts of carotenoid-related compounds. **(B)** The amount of chlorophyll and pheophytin. The data represent three biological replicates. The y-axis represents the concentrations of these compounds as µg Lutein Equivalent gFDW^-1^, and the x-axis displays the genotype names. Error bars denote mean ± SD (n = 3). Statistically significant differences against the wild-type are represented by Student’s t-test as *p* < 0.05 (*).

Given their orthologous relationship with *Arabidopsis thaliana*’s D27, *MpD27–1* and *MpD27–2*, are predicted to function as β-carotene isomerases. This is further supported by the observed increase in transcript abundance of carotenoid associated genes in transcriptome analysis of SL precursor mutants. To investigate whether the loss of gene function impacts carotenoid composition, a targeted UHPLC analysis was conducted on wild-type and mutant genotypes (**Figure 8A**). Additionally, the observed decrease in transcript levels for photosynthesis-related genes, as shown in **Figure 6A**, prompted the quantification of chlorophyll content in the mutants (**Figure 8B**).

The carotenoids violaxanthin, all-*trans*- and *cis*-neoxanthin, lutein, α-carotene and β-carotene have been identified in Marchantia, and several unidentified carotenoids are also present (Takemura et al., 2014). The identification and quantification of carotenoids in the lines studied here showed a significant increase in the levels of *cis*-neoxanthin and lutein across all three SL mutants. Additionally, violaxanthin levels were higher in *Mpccd7*, α-carotene was elevated in *Mpd27–2* and *Mpsmxl*, and β-carotene saw an increase in *Mpd27–1*. An unidentified carotenoid designated as “compound 4”, also exhibited a significant rise in *Mpd27–1* and *Mpd27–2*. Similarly, the chlorophyll data indicated that SL mutants had increased levels of chlorophyll a, b and pheophytin in comparison to the wild-type control (**Figure 8B**).

## Discussion

The strigolactone (SL) pathway is only partially present in Marchantia, and the function of the genes involved is unclear (Walker et al., 2019). In this study, we used CRISPR/Cas9 mutagenesis to understand the roles of SL precursor biosynthesis genes, including *MpD27–1*, *MpD27–2* and *MpCCD7*. The phenotypic comparison of the Marchantia mutants and wild-type plant has elucidated the role of *MpD27–1, MpD27–2* and *MpCCD7* in the release, growth, and germination of gemmae. Analysis of RNA gene expression patterns in these mutants, compared to wild-type plant, revealed an increased transcript abundance of genes involved in terpenoid (e.g., *MpTPS6*) and carotenoid metabolism (e.g., *MpLRAT*, *MpCCD7*, and *BETA-CAROTENE 15,15’-DIOXYGENASE*), as well as *ETHYLENE RESPONSE FACTORS* (e.g., *MpERF10* and *MpERF17*). In contrast, certain genes such as *9-CIS-EPOXYCAROTENOID DIOXYGENASE (MpNCED)*, *EARLY LIGHT INDUCED* (*ELI*), and *LATE EMBRYOGENESIS ABUNDANT (LEA)* showed decreased transcript abundance. Additionally, mutants in SL precursors displayed elevated levels of carotenoids.

### MpD27–1, MpD27–2 and MpCCD7 regulate release of gemmae from the gemmae cup

Bryophytes, which encompass non-vascular plants like liverworts, mosses, and hornworts, can utilize both sexual reproductive organs, such as spores, and vegetative structures like gemmae and some part of the thallus for their propagation (Wyatt, 1982; Chopra and Bhatla, 1990; Buck et al., 2003; Whitaker and Edwards, 2010). Gemmae are small disc-like asexual propagules produced in specialized structures called gemma cups, present on the dorsal side of the thallus (Barnes and Land, 1907, 1908). Raindrops have the potential to expel gemmae from gemma cups (sometimes referred to as ‘splash cups’), allowing plants to grow and establish themselves away from the parental plant (Edwards et al., 2019). The gemmae contained within the gemma cup remain dormant and do not undergo maturation or growth until they are either expelled from the cup or until the maternal plant reaches the end of its life cycle. This suggests a restraint on the liberation and germination of gemmae from the maternal plant. While there is a growing body of information about the genetic factors controlling growth and germination of gemmae (Eklund et al., 2015; Eklund et al., 2018), the genes that regulate the release of gemmae from the cup in liverworts remain elusive.

Here we have shown that defects in functions of *MpD27–1*, *MpD27–2* and *MpCCD7* regulate gemma cup release (**Figures 2**, **3A**). These Marchantia mutants, when compared with wild type, displayed elevated levels of transcripts for *ETHYLENE REPONSE FACTORS* (*ERF*) orthologs, e.g., *Mp7g00860/MpERF10* and *Mp4g22280/MpERF17* (**Figures 6A, D**). The expression of *Mp7g00860/MpERF10* was also validated through ddPCR analysis, providing clear evidence of increased *MpERF10* levels in all three mutants (**Figure 7D**). Furthermore, the MBEX expression tool indicated higher expression of *MpERF17* in gemmae cups (Kawamura et al., 2022). *ERFs*, belonging to the family of AP2/ERF transcription factors, regulate many processes in flowering plants, including seed germination and pod shattering (Pirrello et al., 2006; Chung et al., 2010; Müller and Munné-Bosch, 2015; Chandler and Werr, 2020; Hu et al., 2022; Liu et al., 2022; Ali et al., 2023). If we consider gemmae release to be analogous to seed release, we suggest that the SL biosynthesis genes in Marchantia could act to restrain the function of ERFs, thereby inhibiting the release of gemmae.

### SL precursor biosynthesis genes positively regulate transcript abundance of *ELI*

Mutants of SL precursors exhibited a notable decrease in the transcript abundance of *EARLY LIGHT INDUCED* (*ELI*) genes (**Figure 6A**, **Supplementary Data S1-9**). Specifically, *Mp4g18580* and *Mp4g18590* showed down regulation across all three mutants, while *Mp4g18620* and *Mp4g18610* were down regulated in both *Mpd27–1* and *Mpd27–2*, and *Mp4g18560* experienced a decrease in *Mpd27–2* and *Mpccd7*. Further independent support for the role of these genes in gemma biology came from gene expression profiles. Results obtained using the MBEX expression tool revealed that all four *ELI*/*CAB* genes showed increased expression in the gemma cup (Kawamura et al., 2022). These proteins, while not directly binding chlorophyll (Green et al., 1991), are categorized as part of the CAB family and recognized as early light-induced proteins (ELIPs). ELIPs are triggered by light stress and implicated in pigment biosynthesis and thylakoid membrane assembly, essential for protecting chlorophyll-protein complexes from light damage.

SLs impact the process of senescence by regulating various genetic components involved in photosynthesis (Mayzlish-Gati et al., 2010; Mashiguchi et al., 2021; Li et al., 2022; Schiphorst et al., 2022). For example, the tomato SL biosynthesis *Sl-ORT1* mutant showed reduced chlorophyll content and lower levels of light-harvesting genes (e.g., CAB and RUBISCO) (Mayzlish-Gati et al., 2010). Similarly, the Arabidopsis SL signaling *max2* mutant exhibited delayed senescence and reduced expression of *ELIP1*, *ELIP2*, and *HY5* (Shen et al., 2007). The decreased expression of *ELI* genes in mutants of SL precursors, along with their connection to gemma and gemma cup development, suggests a role for *ELI* in SL-mediated gemmae development. Furthermore, mutants of SL precursors exhibited higher chlorophyll content compared to the control plants, suggesting that SL regulates senescence in Marchantia (**Figure 8B**). However, it is important to note that the increase in chlorophyll content in SL precursor mutants may be attributed to an increase in the number of growing gemmae.

### SL precursor biosynthesis genes suppress germination and growth of gemmae in the dark

Our data show that *MpD27-1*, *MpD27-2*, and *MpCCD7* inhibit the germination and growth of gemmae under dark and nutrient-starved conditions (**Figure 4**, **Supplementary Figure S4**).

This is consistent with other studies on bryophytes that report enhanced tissues growth in mutants defective in the SL and karrikin pathways. For example, SL-deficient (*Ppccd8*) and SL-insensitive (*Ppkai2Lgjm*) moss mutants showed increased extension of moss filaments in the dark (Lopez-Obando et al., 2021). Similarly, karrikin signaling mutants, *Mpkai2a* and *Mpmax2*, showed enhanced growth of gemmae under dark conditions (Mizuno et al., 2021).

SL and ABA are known to interact with each other in regulating plant stress responses. For instance, rice mutants defective in SL production (e.g., *max4*/*dwarf10* and *max3/dwarf17)* and SL perception (e.g., *dwarf3)* showed higher concentrations of ABA (Haider et al., 2018). Similarly, ABA-deficient tomato mutants exhibited reduced concentrations of SL (López-Ráez et al., 2008, 2010). Furthermore, the external application of the SL analog GR24 in *Lotus japonicus* led to an increased transcript abundance of ABA biosynthesis and ABA responsive genes (Liu et al., 2015).

In Marchantia, ABA contributes to the induction of gemmae dormancy. For example, ABA-insensitive Marchantia mutants were unable to establish and maintain gemmae dormancy (Eklund et al., 2018). The reduced expression of ABA-biosynthesis genes, e.g., *9-CIS-EPOXYCAROTENOID DIOXYGENASE* (*MpNCED/Mp2g07800*), and ABA-responsive genes, including *DEHYDRIN* (*MpDHN3*/*Mp6g15610*) and *LATE EMBRYOGENESIS ABUNDANT* (*LEA*/*Mp4g14840*, *Mp4g14870*, *Mp7g06630*, and *Mp4g14880*) (**Figure 7**) in mutants of SL precursors suggests a linkage between SL signaling, ABA production, and the control of gemmae dormancy and germination processes. This conclusion is further supported by evidence from the study by Kawamura et al. (2022), where gene expression profiles generated using the MBEX expression tool indicated that all four *MpLEA* genes were expressed in the gemma cup and sporeling. Additionally, *MpDHN3* and *MpNCED* exhibited higher expression levels in sporelings. Our analysis revealed that *MpNCED* expression was notably decreased in the *Mpccd7* mutant compared to the *Mpd27–1* and *-2* mutants (**Supplementary Data S1-5**). This decrease correlates with enhanced rhizoid production observed in the *Mpccd7* mutant lines (**Supplementary Figure S4**).

### *MpD27–1* positively regulate the transcript abundance of lectin genes

*Mpd7–1* showed reduced transcript abundance of many *LECTIN* genes, specifically A-type and B-type lectins (**Figure 7A**). Notably, gene expression profiles generated using the MBEX expression tool indicated that *Mp8g18650* was highly expressed in gemmae cups (Kawamura et al., 2022). Lectins play a pivotal in establishing a symbiotic relationship between plants and different type of organism under various stress conditions (De Hoff et al., 2009; Jain et al., 2022). For example, under nitrogen stress, legumes secrete lectins from their roots to facilitate bacterial attachment, leading to nodule formation (Downie, 2010). Similarly, certain lectin genes, specifically induced by mycorrhizal associations, are categorized as AM-induced lectin-like genes (Frenzel et al., 2005, 2006). In lichens, an algal-binding lectin has been found to play a key role in establishing a symbiosis between the mycobiont (fungal component) and the photobiont (photosynthetic component) (Nazem-Bokaee et al., 2021).

SLs are crucial plant hormones that regulate plant architecture and act as signaling molecules exuded into the rhizosphere to foster symbiotic relationships, specifically with AM fungi. Although various species of Marchantia, including *M. pappeana* and *M. paleacea*, interact with AM fungi, *M. polymorpha* stands out as it does not form association with AM fungi. Despite this *M. polymorpha* possesses the capability to interact with a wide range of microbial communities (Poveda, 2020). For example, *M. polymorpha* was able to establish interactions with various pathogenic and beneficial endophytic fungi (Nelson et al., 2018; Nelson and Shaw, 2019). Similarly, *M. polymorpha* and *M. paleacea* interacted with diverse bacterial genera known for plant-growth promotion, exudate degradation, nitrogen fixation and disease-suppression (Van Damme et al., 2008; Alcaraz et al., 2018).

The observed decrease in lectin genes expression in the SL precursor mutant *Mpd27–1* suggests that *MpD27–1* might be involved in establishing interactions with microorganisms through lectins during biotic and abiotic stress responses.

### SL precursor biosynthesis mutants of Marchantia showed higher transcripts abundance of carotenoid-related genes

In SL precursor biosynthesis mutants, we observed an increased transcript abundance of carotenoid related genes including *LRAT* (*LECITHIN RETINOL ACYLTRANSFERASE*) and *TS* (*TERPENE SYNTHASE*) (**Figure 6**, **Supplementary Data S1-9**). β-carotene is cleaved into two molecules of retinal, which can then be reduced to retinol (vitamin A) (D’Ambrosio et al., 2011). LRAT plays a crucial role in metabolism of vitamin A by catalysing the formation of fatty acid retinyl esters from all-trans-retinol (Sears and Palczewski, 2016). The direct functional counterparts of LRAT are absent in plants, as plants do not require the synthesis of retinol. However, through a sequence-based homology analysis, homologues of LRAT have been identified in plants and are known as the H-Box/NC domain-containing gene family (Kaloudas and Penchovsky, 2018). The roles of LRAT homologs in plants still need investigation. However in Arabidopsis, NC domain containing proteins including *AT5G06370*, *AT3G02700*, and *AT5G06370* regulate pollen germination and pollen tube growth (Wang et al., 2008). The higher abundance of *LRAT* homologs in Marchantia SL mutants aligns with their role in enhancing the germination of reproductive structures in Arabidopsis, which, in this study are gemmae. This suggests a conservation of certain functional domains across species, despite the differing metabolic requirements between plants and animals.

Plant terpene synthases (TS) are used to make monoterpenes, sesquiterpenes, and diterpenes that are then utilized in the production of ABA, chlorophyll and carotenoids (Cheng et al., 2007). TS are responsible for converting simple hydrocarbon skeletons into the vast array of terpenes important in plant defense, nodule formation, pollinator attraction, and interplant communication (Huang and Osbourn, 2019). For example, over expression of some terpenoid and terpene synthesis genes from *Salvia officinalis* in soybean are known to modulate rhizobia interaction and nodulation (Ali et al., 2021). The observed increase in *LRAT* and *TS* transcripts in SL precursor biosynthesis mutants may be attributed to either compensation for reduced metabolites controlled by SL precursor biosynthesis genes or because of a feedback mechanism where disrupted SL synthesis affects precursor synthesis. Additionally, *Mpd27–2* had significantly higher levels of *MpCCD7* (**Figure 7B**) suggesting that MpD27–2 and MpCCD7 both belong to the same metabolic pathway. When upstream components of a metabolic pathway are compromised then the amount of downstream enzyme could increase due to feedback regulation, compensation mechanisms, regulatory signals, or availability of substrate.

### Disruption of SL precursor biosynthesis genes altered carotenoid profiling in Marchantia

Alongside elevated expression of *TS* and *LRAT*, *Mpd27–1, Mpd27–2* and *Mpccd7* mutants exhibited an increased concentration of carotenoid-related metabolites (**Figure 8A**). Each of the three mutants showed a marked increase in the levels of *cis*-neoxanthin and lutein. The increased *cis*-neoxanthin could be attributed to downregulation of *MpNCED* in the SL mutants (**Supplementary Data S1-9**). NCED plays a pivotal role in ABA biosynthesis, converting 9-*cis*-violaxanthin and 9′-*cis*-neoxanthin into xanthoxin, an ABA precursor (Perreau et al., 2020). Therefore, a decrease in *MpNCED* abundance could naturally result in the accumulation of neoxanthin, as less of it is converted into xanthoxin. Furthermore, the observed increase in lutein levels may also be attributed to disruptions in the activity of SL precursor biosynthesis enzymes, specifically those involved in cleaving 9-*cis*-zeaxanthin and 9-*cis*-lutein. For example, an *in vitro* study involving CCD7 enzymes sourced from rice, Arabidopsis, and garden pea demonstrated CCD7’s broad specificity. Notably, AtCCD7 and PsCCD7 were shown to effectively catalyze the conversion of 9-*cis*-lutein into 9-*cis*-3-OH-ϵ-apo-10′-carotenal (Baz, 2018). This evidence underscores CCD7’s role in carotenoid metabolism and its potential impact on lutein accumulation when the normal function of SL precursor biosynthesis enzymes is disrupted.

*Mpd27–1* and *Mpd27–2* mutants were observed to have elevated levels of β-carotene and α-carotene, respectively. This rise in carotenoid content within these mutants could be attributed to a decrease in isomerization processes, preventing their usual conversion into other compounds. The D27 enzyme from rice has been demonstrated *in vitro* to catalyze reactions across a variety of bicyclic carotenoid substrates, each containing at least one unsubstituted β-ionone ring, e.g., β-carotene, α-carotene, and cryptoxanthin (Bruno and Al-Babili, 2016). Similarly, AtD27 was capable of catalyzing the reverse isomerization of all-trans-/9-*cis*-β-carotene, showcasing enzyme activity that selectively targets specific isomeric forms (Abuauf et al., 2018). Notably, neither the rice nor the Arabidopsis D27 enzyme exhibits isomerization activity with 13-*cis*- or 15-*cis*-β-carotene, underscoring a distinct specificity for the C9-C10 double bond in these carotenoids. This specificity may play a critical role in the observed accumulation of β- and α-carotene in the *d27* mutants, as it suggests a limited ability of the D27 enzyme to process these carotenoids into other forms, thereby contributing to their increased levels.

*Mpd27–1* and *Mpd27–2* both exhibited elevated levels of an unidentified carotenoid compound (**Figure 8A**). This observation suggests that MpD27–1 and MpD27–2 might play roles in the metabolism of carotenoid-like compounds, potentially beyond their established functions in carotene catabolism. Our hypothesis is further supported by a study where the enzymatic action of OsD27 on all-trans-α-carotene might be involved in producing a SL like compound such as heliolactone (Bruno and Al-Babili, 2016).

In this context, we propose that both MpD27–1 and MpD27–2, SL pathway isomerases have specific regulatory functions in the synthesis of SL compounds originating from β-carotene and α-carotene, respectively. This specific role of the two enzymes was evolutionarily favored to produce a diverse range of carotenoid-derived compounds, which might be implicated in controlling plant developmental processes such as regulation of release, germination, and growth of gemmae. Such a mechanism ensures that germination occurs under favorable growth conditions, which include sufficient ambient light and water availability.

## Summary

Understanding the role of plant pigments like carotenoids and chlorophylls is vital to gain insights into the remarkable ability of plants to adapt to diverse environmental niches (Boncan et al., 2020). Our study has demonstrated that disruptions in the functioning of SL precursor biosynthesis genes, such as *MpD27–1*, *MpD27–2*, and *MpCCD7*, led to the following outcomes: an increase in gemma release, enhanced germination of gemma when incubated in darkness, reduced transcript levels of genes related to photosynthesis and stress, elevated levels of genes related to ethylene and carotenoids, and an increased abundance of carotenoids. Considering these results, we propose that SL precursor biosynthesis genes have specific regulatory functions in the synthesis of SL compounds. This specificity of SL enzymes was evolutionarily favored to produce a diverse range of carotenoid-derived compounds, which might be implicated in controlling plant developmental processes, such as the regulation of release, germination, and growth of gemmae. Such a mechanism ensures that germination occurs only under favorable growth conditions, which include sufficient ambient light and water availability.

## Data availability statement

The data presented in the study are deposited in the BioProject database, NCBI repository, accession numbers PRJNA1123149, SAMN41798178, SAMN41798179, SAMN41798180, SAMN41798181, SAMN41798182, SAMN41798183, SAMN41798184, SAMN41798185, SAMN41798186, SAMN41798187, and SAMN41798188, SAMN41798189.

## Author contributions

RJ: Conceptualization, Data curation, Formal Analysis, Funding acquisition, Methodology, Project administration, Validation, Writing – original draft, Writing – review & editing. JT: Data curation, Formal Analysis, Software, Writing – review & editing. CA: Methodology, Writing – review & editing. BJ: Methodology, Writing – review & editing. RD: Methodology, Writing – review & editing. NA: Methodology, Writing – review & editing. YZ: Methodology, Writing – review & editing. KD: Conceptualization, Writing – review & editing. KS: Conceptualization, Writing – review & editing.

## Supplementary material

Supplementary Figure 1 CRISPR/Cas9-generated strigolactones mutants of Marchantia. DNA sequence of CRISPR-induced mutations in *MpD27–1* (*Mp6g03970*), *MpD27–2* (*Mp6g01750*), *MpCCD7* (*Mp2g03280*) and *MpSMXL (Mp3g06310).* The underlined sequences enclosed by large black brackets represent the guides used, the symbol “—” represents the deletion, and the symbol “+ “represents insertions. The purple boxes represent exons, whereas the black lines represent the introns.

Supplementary Figure 2
**(A)** Phenotypes of wild-type and *Mpsmxl#11, Mpd27–1#16*, *Mpd27–2#9* and *Mpccd7#17* mutant plants. Plants were grown on complete media (1/2 strength Gamborg’s B5 medium supplemented with 1% sucrose and solidified with 1% agar) for 10 weeks at 25°C, 16-hour photoperiod and a light intensity of 30 mol m^-2^ s^-1^, provided by cool fluorescent tubes. **(B)** Total number of gemmae in GC located at different positions across the thallus for the wild-type genotype. The GC positions were numbered as shown in .

Supplementary Figure 3 Dormant gemmae of *Mpsmxl#11, MpD27–1, MpD27–2, MpCCD7* and wild-type were grown on carbon- and nutrient-starved medium under light for 10 days. **(A)** Phenotypes of gemmae, where images were taken with a light microscope. **(B)** Area of gemmae, which was measured by image J. Error bars denote mean ± SD (n = 50). The Student’s test was employed to compare mutant lines with wild-type. Statistically significant differences against both wild-types are represented at *p* < 0.05 (*).

Supplementary Figure 4 Dormant gemmae of *Mpsmxl#11*, *MpD27–1, MpD27–2, MpCCD7* and wild-type were grown on carbon- and nutrient-starved medium in the complete darkness for 10 days. The graph represents the percentage of gemmae with rhizoids. Scale bars represent 1 mm, images taken with a light microscope. Error bars denote mean ± SD (n = 50). The Student’s t-test was used to compare mutant lines with wild-type. Statistically significant differences against the wild-type are represented as *p* < 0.05 (*).

Supplementary Figure 5 Transcriptome analysis of *Mpd27–1*, *Mpd27–2* and *Mpccd7* and wild-type genotype. A dendrogram generated by hierarchical clustering illustrating the relationships between the various gene expression profiles. Hierarchical clustering performed on differentially expressed genes defined by ANOVA with a FDR <0.05. Using all replicates per group, top 2000 most variable genes were clustered. The scale bar represents relative expression ±2 SD from the mean.

## References

[B1] Abuauf H. Haider I. Jia K.-P. Ablazov A. Mi J. Blilou I.. (2018). The arabidopsis dwarf27 gene encodes an all-trans-/9-cis-B-carotene isomerase and is induced by auxin, abscisic acid and phosphate deficiency. Plant Sci. 277, 33–42. doi: 10.1016/j.plantsci.2018.06.024 30466598

[B2] Albert N. W. Thrimawithana A. H. Mcghie T. K. Clayton W. A. Deroles S. C. Schwinn K. E.. (2018). Genetic analysis of the liverwort marchantia polymorpha reveals that R2r3 myb activation of flavonoid production in response to abiotic stress is an ancient character in land plants. New Phytol. 218, 554–566. doi: 10.1111/nph.15002 29363139

[B3] Alcaraz L. D. Peimbert M. Barajas H. R. Dorantes-Acosta A. E. Bowman J. L. Arteaga-Vázquez M. A. (2018). Marchantia liverworts as A proxy to plants’ Basal microbiomes. Sci. Rep. 8, 12712. doi: 10.1038/s41598-018-31168-0 30140076 PMC6107579

[B4] Ali M. Miao L. Darwish D. B. Alrdahe S. S. Benedito V. A. Tadege M.. (2021). Overexpression of terpenoid biosynthesis genes from garden sage (Salvia officinalis) modulates rhizobia interaction and nodulation in soybean. Front. In Plant Sci. 12, 783269. doi: 10.3389/fpls.2021.783269 35003167 PMC8733304

[B5] Ali S. Kucek L. K. Riday H. Krom N. Krogman S. Cooper K.. (2023). Transcript profiling of hairy vetch (Vicia villosa roth) identified interesting genes for seed dormancy. Plant Genome 16 (2), E20330. doi: 10.1002/tpg2.20330 37125613 PMC12806872

[B6] Aquino B. Bradley J. M. Lumba S. (2021). On the outside looking in: roles of endogenous and exogenous strigolactones. Plant J. 105, 322–334. doi: 10.1111/tpj.15087 33215770

[B7] Barnes C. R. Land W. (1908). Bryological papers. Ii. The origin of the cupule of marchantia. Botanic. Gazette 46, 401–409. doi: 10.1086/329782

[B8] Barnes C. R. Land W. J. G. (1907). Bryological papers. I. The origin of air chambers. Botanic. Gazette 44, 197–213. doi: 10.1086/329317

[B9] Baz L. (2018). Biosynthesis Of Carotenoid-Derived Plant Signaling Molecules.

[B10] Bidartondo M. I. Read D. J. Trappe J. M. Merckx V. Ligrone R. Duckett J. G. (2011). The dawn of symbiosis between plants and fungi. Biol. Lett. 7, 574–577. doi: 10.1098/rsbl.2010.1203 21389014 PMC3130224

[B11] Boncan D. A. T. Tsang S. S. K. Li C. Lee I. H. T. Lam H. M. Chan T. F.. (2020). Terpenes and terpenoids in plants: interactions with environment and insects. Int. J. Mol. Sci. 21 (19), 7382. doi: 10.3390/ijms21197382 33036280 PMC7583029

[B12] Bowman J. L. Arteaga-Vazquez M. Berger F. Briginshaw L. N. Carella P. Aguilar-Cruz A.. (2022). The renaissance and enlightenment of marchantia as A model system. Plant Cell 34, 3512–3542. doi: 10.1093/plcell/koac219 35976122 PMC9516144

[B13] Brundrett M. C. (2002). Coevolution of roots and mycorrhizas of land plants. New Phytol. 154, 275–304. doi: 10.1046/j.1469-8137.2002.00397.x 33873429

[B14] Bruno M. Al-Babili S. (2016). On the substrate specificity of the rice strigolactone biosynthesis enzyme dwarf27. Planta 243, 1429–1440. doi: 10.1007/s00425-016-2487-5 26945857

[B15] Buck W. R. Allen B. Pursell R. A. (2003). Recent literature on bryophytes. Bryologist 106 (2), 332–340. doi: 10.1639/0007-2745(2003)106[0332:RLOB]2.0.CO;2

[B16] Carella P. Schornack S. (2018). Manipulation of bryophyte hosts by pathogenic and symbiotic microbes. Plant And Cell Physiol. 59, 656–665. doi: 10.1093/pcp/pcx182 PMC6018959 29177478

[B17] Chandler J. Werr W. (2020). A phylogenetically conserved APETALA2/ethylene response factor, ERF12, regulates arabidopsis floral development. Plant Mol. Biol. 102, 39–54. doi: 10.1007/s11103-019-00936-5 31807981 PMC6976583

[B18] Cheng A. X. Lou Y. G. Mao Y. B. Lu S. Wang L. J. Chen X. Y. (2007). Plant terpenoids: biosynthesis and ecological functions. J. Integr. Plant Biol. 49, 179–186. doi: 10.1111/j.1744-7909.2007.00395.x

[B19] Chopra R. Bhatla S. C. (1990). Bryophyte development: physiology and biochemistry (CRC Press).

[B20] Chung M. Y. Vrebalov J. Alba R. Lee J. Mcquinn R. Chung J. D.. (2010). A tomato (Solanum lycopersicum) APETALA2/ERF gene, SlAP2a, is a negative regulator of fruit ripening. Plant J. 64, 936–947. doi: 10.1111/tpj.2010.64.issue-6 21143675

[B21] Costa D. P. Peralta D. F. (2015). Bryophytes diversity in Brazil. Rodriguésia 66, 1063–1071. doi: 10.1590/2175-7860201566409

[B22] D’Ambrosio D. N. Clugston R. D. Blaner W. S. (2011). Vitamin A metabolism: an update. Nutrients 3, 63–103. doi: 10.3390/nu3010063 21350678 PMC3042718

[B23] Dang Y. Jia G. Choi J. Ma H. Anaya E. Ye C.. (2015). Optimizing sgRNA structure to improve CRISPR-Cas9 knockout efficiency. Genome Biol. 16, 1–10. doi: 10.1186/s13059-015-0846-3 26671237 PMC4699467

[B24] De Hoff P. L. Brill L. M. Hirsch A. M. (2009). Plant lectins: the ties that bind in root symbiosis and plant defense. Mol. Genet. Genomics 282, 1–15. doi: 10.1007/s00438-009-0460-8 19488786 PMC2695554

[B25] Delaux P.-M. Radhakrishnan G. V. Jayaraman D. Cheema J. Malbreil M. Volkening J. D.. (2015). Algal ancestor of land plants was preadapted for symbiosis. Proc. Natl. Acad. Sci. 112, 13390–13395. doi: 10.1073/pnas.1515426112 26438870 PMC4629359

[B26] Delaux P.-M. Xie X. Timme R. E. Puech-Pages V. Dunand C. Lecompte E.. (2012). Origin of strigolactones in the green lineage. New Phytol. 195, 857–871. doi: 10.1111/j.1469-8137.2012.04209.x 22738134

[B27] de Saint Germain A. Bonhomme S. Boyer F.-D. Rameau C. (2013). Novel insights into strigolactone distribution and signalling. Curr. Opin. Plant Biol. 16, 583–589. doi: 10.1016/j.pbi.2013.06.007 23830996

[B28] Doench J. G. Hartenian E. Graham D. B. Tothova Z. Hegde M. Smith I.. (2014). Rational design of highly active sgRNAs for CRISPR-Cas9–mediated gene inactivation. Nat. Biotechnol. 32, 1262–1267. doi: 10.1038/nbt.3026 25184501 PMC4262738

[B29] Downie J. A. (2010). The roles of extracellular proteins, polysaccharides and signals in the interactions of rhizobia with legume roots. FEMS Microbiol. Rev. 34, 150–170. doi: 10.1111/j.1574-6976.2009.00205.x 20070373

[B30] Drummond R. S. Janssen B. J. Luo Z. Oplaat C. Ledger S. E. Wohlers M. W.. (2015). Environmental control of branching in petunia. Plant Physiol. 168, 735–751. doi: 10.1104/pp.15.00486 25911529 PMC4453797

[B31] Drummond R. S. Lee H. W. Luo Z. Janssen B. J. Snowden K. C. (2023). Varying the expression pattern of the strigolactone receptor gene DAD2 results in phenotypes distinct from both wild type and knockout mutants. Front. Plant Sci. 14, 1277617. doi: 10.3389/fpls.2023.1277617 37900765 PMC10600376

[B32] Dun E. A. De Saint Germain A. Rameau C. Beveridge C. A. (2013). Dynamics of strigolactone function and shoot branching responses in Pisum sativum. Mol. Plant 6, 128–140. doi: 10.1093/mp/sss131 23220942

[B33] Edgar R. C. (2004). MUSCLE: multiple sequence alignment with high accuracy and high throughput. Nucleic Acids Res. 32, 1792–1797. doi: 10.1093/nar/gkh340 15034147 PMC390337

[B34] Edwards J. Laskowski M. Baskin T. Mitchell N. Demeo B. (2019). The role of water in fast plant movements. Integr. Comp. Biol. 59, 1525–1534. doi: 10.1093/icb/icz081 31168592

[B35] Eklund D. M. Ishizaki K. Flores-Sandoval E. Kikuchi S. Takebayashi Y. Tsukamoto S.. (2015). Auxin produced by the indole-3-pyruvic acid pathway regulates development and gemmae dormancy in the liverwort Marchantia polymorpha. Plant Cell 27, 1650–1669. doi: 10.1105/tpc.15.00065 26036256 PMC4498201

[B36] Eklund D. M. Kanei M. Flores-Sandoval E. Ishizaki K. Nishihama R. Kohchi T.. (2018). An evolutionarily conserved abscisic acid signaling pathway regulates dormancy in the liverwort Marchantia polymorpha. Curr. Biol. 28, 3691–3699. e3. doi: 10.1016/j.cub.2018.10.018 30416060

[B37] Foo E. Reid J. B. (2013). Strigolactones: new physiological roles for an ancient signal. J. Plant Growth Regul. 32, 429–442. doi: 10.1007/s00344-012-9304-6

[B38] Frenzel A. Manthey K. Perlick A. M. Meyer F. Pühler A. Küster H.. (2005). Combined transcriptome profiling reveals a novel family of arbuscular mycorrhizal-specific Medicago truncatula lectin genes. Mol. Plant-Microbe Interact. 18, 771–782. doi: 10.1094/MPMI-18-0771 16134889

[B39] Frenzel A. Tiller N. Hause B. Krajinski F. (2006). The conserved arbuscular mycorrhiza-specific transcription of the secretory lectin mtlec 5 is mediated by A short upstream sequence containing specific protein binding sites. Planta 224, 792–800. doi: 10.1007/s00425-006-0262-8 16596411

[B40] Ge S. X. Son E. W. Yao R. (2018). Idep: an integrated web application for differential expression and pathway analysis of rna-seq data. BMC Bioinf. 19, 1–24. doi: 10.1186/s12859-018-2486-6 PMC6299935 30567491

[B41] Gomez-Roldan V. Fermas S. Brewer P. B. Puech-Pagès V. Dun E. A. Pillot J.-P.. (2008). Strigolactone inhibition of shoot branching. Nature 455, 189–194. doi: 10.1038/nature07271 18690209

[B42] Green B. R. Pichersky E. Kloppstech K. (1991). Chlorophyll A/B-binding proteins: an extended family. Trends In Biochem. Sci. 16, 181–186. doi: 10.1016/0968-0004(91)90072-4 1882419

[B43] Guindon S. Gascuel O. (2003). A simple, fast, and accurate algorithm to estimate large phylogenies by maximum likelihood. System. Biol. 52, 696–704. doi: 10.1080/10635150390235520 14530136

[B44] Haider I. Andreo-Jimenez B. Bruno M. Bimbo A. Floková K. Abuauf H.. (2018). The interaction of strigolactones with abscisic acid during the drought response in rice. J. Exp. Bot. 69, 2403–2414. doi: 10.1093/jxb/ery089 29538660

[B45] Hu Y. Han Z. Wang T. Li H. Li Q. Wang S.. (2022). Ethylene response factor MdERF4 and histone deacetylase MdHDA19 suppress apple fruit ripening through histone deacetylation of ripening-related genes. Plant Physiol. 188, 2166–2181. doi: 10.1093/plphys/kiac016 35088866 PMC8968277

[B46] Huang A. C. Osbourn A. (2019). Plant terpenes that mediate below-ground interactions: prospects for bioengineering terpenoids for plant protection. Pest Manage. Sci. 75, 2368–2377. doi: 10.1002/ps.5410 PMC6690754 30884099

[B47] Humphreys C. P. Franks P. J. Rees M. Bidartondo M. I. Leake J. R. Beerling D. J. (2010). Mutualistic mycorrhiza-like symbiosis in the most ancient group of land plants. Nat. Commun. 1, 103. doi: 10.1038/ncomms1105 21045821

[B48] Ishizaki K. Nishihama R. Yamato K. T. Kohchi T. (2016). Molecular genetic tools and techniques for Marchantia polymorpha research. Plant Cell Physiol. 57, 262–270. doi: 10.1093/pcp/pcv097 26116421

[B49] Jain M. Amera G. M. Muthukumaran J. Singh A. K. (2022). Insights into biological role of plant defense proteins: A review. Biocatal. Agric. Biotechnol. 40, 102293. doi: 10.1016/j.bcab.2022.102293

[B50] Kaloudas D. Penchovsky R. (2018). Arabidopsis homologues to the LRAT a possible substrate for new plant-based anti-cancer drug development. Int. J. Biomed. Clin. Eng. (IJBCE) 7, 40–52. doi: 10.4018/IJBCE

[B51] Kawamura S. Romani F. Yagura M. Mochizuki T. Sakamoto M. Yamaoka S. (2022). MarpolBase expression: A web-based, comprehensive platform for visualization and analysis of transcriptomes in the liverwort Marchantia polymorpha. Plant Cell Physiol. 63, 1745–1755. 36083565 10.1093/pcp/pcac129 PMC9680858

[B52] Kim D. Paggi J. M. Park C. Bennett C. Salzberg S. L. (2019). Graph-based genome alignment and genotyping with HISAT2 and HISAT-genotype. Nat. Biotechnol. 37, 907–915. doi: 10.1038/s41587-019-0201-4 31375807 PMC7605509

[B53] Kodama K. Rich M. K. Yoda A. Shimazaki S. Xie X. Akiyama K.. (2022). An ancestral function of strigolactones as symbiotic rhizosphere signals. Nat. Commun. 13, 3974. doi: 10.1038/s41467-022-31708-3 35803942 PMC9270392

[B54] Kohchi T. Yamato K. T. Ishizaki K. Yamaoka S. Nishihama R. (2021). Development and molecular genetics of Marchantia polymorpha. Annu. Rev. Plant Biol. 72, 677–702. doi: 10.1146/annurev-arplant-082520-094256 33684298

[B55] Komatsu A. Kodama K. Mizuno Y. Fujibayashi M. Naramoto S. Kyozuka J. (2023). Control of vegetative reproduction in Marchantia polymorpha by the KAI2-ligand signaling pathway. Curr. Biol. 33, 1196–1210. e4. doi: 10.1016/j.cub.2023.02.022 36863344

[B56] Li F.-W. Nishiyama T. Waller M. Frangedakis E. Keller J. Li Z.. (2020). Anthoceros genomes illuminate the origin of land plants and the unique biology of hornworts. Nat. Plants 6, 259–272. doi: 10.1038/s41477-020-0618-2 32170292 PMC8075897

[B57] Li Z. Pi Y. Zhai C. Xu D. Ma W. Chen H.. (2022). The strigolactone receptor SlDWARF14 plays a role in photosynthetic pigment accumulation and photosynthesis in tomato. Plant Cell Rep. 41, 2089–2105. doi: 10.1007/s00299-022-02908-4 35907035

[B58] Liao Y. Smyth G. K. Shi W. (2014). featureCounts: an efficient general purpose program for assigning sequence reads to genomic features. Bioinformatics 30, 923–930. doi: 10.1093/bioinformatics/btt656 24227677

[B59] Liu J. W. He H. Z. Vitali M. Visentin I. Charnikhova T. Haider I.. (2015). Osmotic stress represses strigolactone biosynthesis in roots: exploring the interaction between strigolactones and ABA under abiotic stress. Planta 241, 1435–1451. doi: 10.1007/s00425-015-2266-8 25716094

[B60] Liu J. Zhang Y. Jiang Y. Sun H. Duan R. Qu J.. (2022). Formation mechanism and occurrence law of pod shattering in soybean: A review. Phyton 91. doi: 10.32604/phyton.2022.019870

[B61] Lopez-Obando M. Guillory A. Boyer F.-D. Cornu D. Hoffmann B. Le Bris P.. (2021). The Physcomitrium (Physcomitrella) patens PpKAI2L receptors for strigolactones and related compounds function via MAX2-dependent and-independent pathways. Plant Cell 33, 3487–3512. doi: 10.1093/plcell/koab217 34459915 PMC8662777

[B62] López-Ráez J. A. Charnikhova T. Gómez-Roldán V. Matusova R. Kohlen W. De Vos R.. (2008). Tomato strigolactones are derived from carotenoids and their biosynthesis is promoted by phosphate starvation. New Phytol. 178, 863–874. doi: 10.1111/j.1469-8137.2008.02406.x 18346111

[B63] López-Ráez J. A. Kohlen W. Charnikhova T. Mulder P. Undas A. K. Sergeant M. J.. (2010). Does abscisic acid affect strigolactone biosynthesis? New Phytol. 187, 343–354. doi: 10.1111/j.1469-8137.2010.03291.x 20487312

[B64] Mashiguchi K. Seto Y. Yamaguchi S. (2021). Strigolactone biosynthesis, transport and perception. Plant J. 105, 335–350. doi: 10.1111/tpj.15059 33118266

[B65] Mayzlish-Gati E. Lekkala S. P. Resnick N. Wininger S. Bhattacharya C. Lemcoff J. H.. (2010). Strigolactones are positive regulators of light-harvesting genes in tomato. J. Exp. Bot. 61, 3129–3136. doi: 10.1093/jxb/erq138 20501744 PMC2892153

[B66] Mizuno Y. Komatsu A. Shimazaki S. Naramoto S. Inoue K. Xie X.. (2021). Major components of the KARRIKIN INSENSITIVE2-dependent signaling pathway are conserved in the liverwort Marchantia polymorpha. Plant Cell 33, 2395–2411. doi: 10.1093/plcell/koab106 33839776 PMC8364241

[B67] Morris J. L. Puttick M. N. Clark J. W. Edwards D. Kenrick P. Pressel S.. (2018). The timescale of early land plant evolution. Proc. Natl. Acad. Sci. 115, E2274–E2283. doi: 10.1073/pnas.1719588115 29463716 PMC5877938

[B68] Müller M. Munné-Bosch S. (2015). Ethylene response factors: a key regulatory hub in hormone and stress signaling. Plant Physiol. 169, 32–41. doi: 10.1104/pp.15.00677 26103991 PMC4577411

[B69] Nazem-Bokaee H. Hom E. F. Warden A. C. Mathews S. Gueidan C. (2021). Towards a systems biology approach to understanding the lichen symbiosis: opportunities and challenges of implementing network modelling. Front. Microbiol. 12, 667864. doi: 10.3389/fmicb.2021.667864 34012428 PMC8126723

[B70] Nelson J. M. Hauser D. A. Hinson R. Shaw A. J. (2018). A novel experimental system using the liverwort Marchantia polymorpha and its fungal endophytes reveals diverse and context-dependent effects. New Phytol. 218, 1217–1232. doi: 10.1111/nph.15012 29411387

[B71] Nelson J. Shaw A. J. (2019). Exploring the natural microbiome of the model liverwort: fungal endophyte diversity in Marchantia polymorpha L. Symbiosis 78, 45–59. doi: 10.1007/s13199-019-00597-4

[B72] Perreau F. Frey A. Effroy-Cuzzi D. Savane P. Berger A. Gissot L.. (2020). ABSCISIC ACID-DEFICIENT4 has an essential function in both cis-violaxanthin and cis-neoxanthin synthesis. Plant Physiol. 184, 1303–1316. doi: 10.1104/pp.20.00947 32883757 PMC7608147

[B73] Pirrello J. Jaimes-Miranda F. Sanchez-Ballesta M. T. Tournier B. Khalil-Ahmad Q. Regad F.. (2006). Sl-ERF2, a tomato ethylene response factor involved in ethylene response and seed germination. Plant Cell Physiol. 47, 1195–1205. doi: 10.1093/pcp/pcj084 16857696

[B74] Poveda J. (2020). Marchantia polymorpha as a model plant in the evolutionary study of plant-microorganism interactions. Curr. Plant Biol. 23, 100152. doi: 10.1016/j.cpb.2020.100152

[B75] Robinson M. D. Mccarthy D. J. Smyth G. K. (2010). edgeR: a Bioconductor package for differential expression analysis of digital gene expression data. bioinformatics 26, 139–140. doi: 10.1093/bioinformatics/btp616 19910308 PMC2796818

[B76] Schiphorst C. Achterberg L. Gómez R. Koehorst R. Bassi R. Van Amerongen H.. (2022). The role of light-harvesting complex I in excitation energy transfer from LHCII to photosystem I in Arabidopsis. Plant Physiol. 188, 2241–2252. doi: 10.1093/plphys/kiab579 34893885 PMC8968287

[B77] Schneider C. A. Rasband W. S. Eliceiri K. W. (2012). NIH Image to ImageJ: 25 years of image analysis. Nat. Methods 9, 671–675. doi: 10.1038/nmeth.2089 22930834 PMC5554542

[B78] Sears A. E. Palczewski K. (2016). Lecithin: retinol acyltransferase: a key enzyme involved in the retinoid (visual) cycle. Biochemistry 55, 3082–3091. doi: 10.1021/acs.biochem.6b00319 27183166 PMC5555363

[B79] Sgroi M. Paszkowski U. (2020). Transcriptional responses to arbuscular mycorrhizal symbiosis development are conserved in the early divergent Marchantia paleacea. BioRxiv, 422721. doi: 10.1101/2020.12.14.422721

[B80] Shaw A. J. Szövényi P. Shaw B. (2011). Bryophyte diversity and evolution: windows into the early evolution of land plants. Am. J. Bot. 98, 352–369. doi: 10.3732/ajb.1000316 21613131

[B81] Shen H. Luong P. Huq E. (2007). The F-box protein MAX2 functions as a positive regulator of photomorphogenesis in Arabidopsis. Plant Physiol. 145, 1471–1483. doi: 10.1104/pp.107.107227 17951458 PMC2151697

[B82] Sun T. Rao S. Zhou X. Li L. (2022). Plant carotenoids: recent advances and future perspectives. Mol. Horticult. 2, 3. doi: 10.1186/s43897-022-00023-2 PMC10515021 37789426

[B83] Takemura M. Maoka T. Misawa N. (2014). Carotenoid analysis of a liverwort Marchantia polymorpha and functional identification of its lycopene β-and ϵ-cyclase genes. Plant Cell Physiol. 55, 194–200. doi: 10.1093/pcp/pct170 24285752

[B84] Van Damme E. J. Lannoo N. Peumans W. J. (2008). “Plant lectins,” in Advances in botanical research (Elsevier). doi: 10.1016/S0065-2296(08)00403-5

[B85] Walker C. H. Siu-Ting K. Taylor A. O’connell M. J. Bennett T. (2019). Strigolactone synthesis is ancestral in land plants, but canonical strigolactone signalling is a flowering plant innovation. BMC Biol. 17, 1–19. doi: 10.1186/s12915-019-0689-6 31488154 PMC6728956

[B86] Wang Y. Zhang W.-Z. Song L.-F. Zou J.-J. Su Z. Wu W.-H. (2008). Transcriptome analyses show changes in gene expression to accompany pollen germination and tube growth in Arabidopsis. Plant Physiol. 148, 1201–1211. doi: 10.1104/pp.108.126375 18775970 PMC2577266

[B87] Whitaker D. L. Edwards J. (2010). Sphagnum moss disperses spores with vortex rings. Science 329, 406–406. doi: 10.1126/science.1190179 20651145

[B88] Wyatt R. (1982). Population ecology of bryophytes. J. Hattori Botanic. Lab. 52, 179–198. doi: 10.18968/jhbl.52.0_179

[B89] Yeum K.-J. Russell R. M. (2002). Carotenoid bioavailability and bioconversion. Annu. Rev. Nutr. 22, 483–504. doi: 10.1146/annurev.nutr.22.010402.102834 12055355

