# Supplementary material for: DWARF27 and CAROTENOID CLEAVAGE DIOXYGENASE 7 genes regulate release, germination and growth of gemma in Marchantia polymorpha
Source: Front Plant Sci. 2024 Jun 25;15:1358745. doi: 10.3389/fpls.2024.1358745 (PMC11231376; doi:10.3389/fpls.2024.1358745)

Supplementary Figure S1

MpD27-1 CRISPR/Cas9 mutants

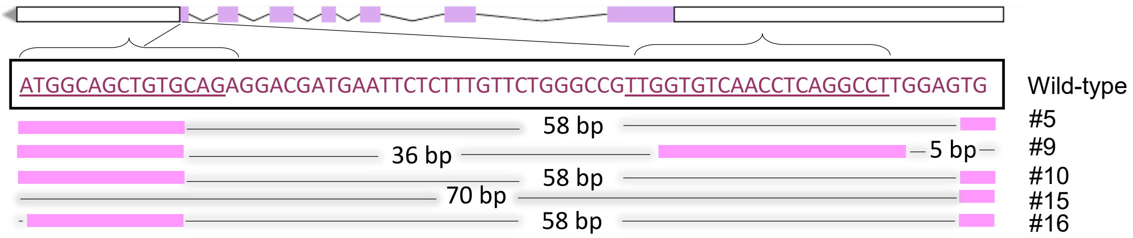

MpD27-2 CRISPR/Cas9 mutants

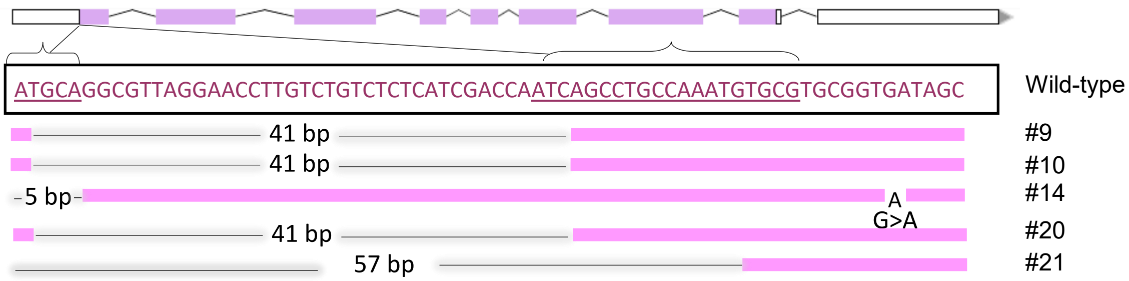

MpCCD7 CRISPR/Cas9 mutants

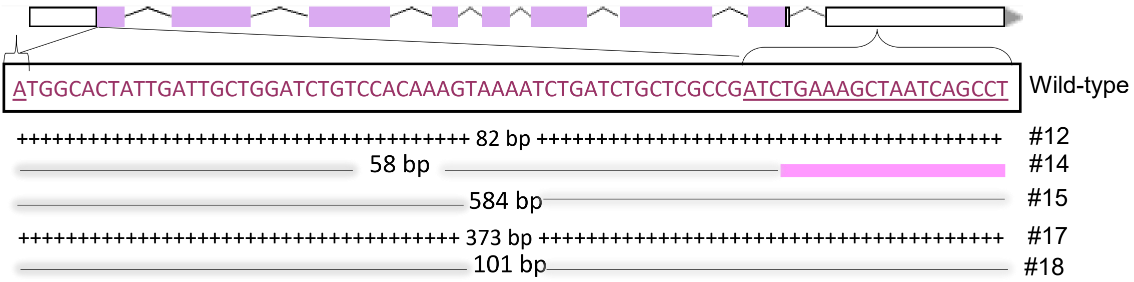

MpSMXL CRISPR/Cas9 mutant

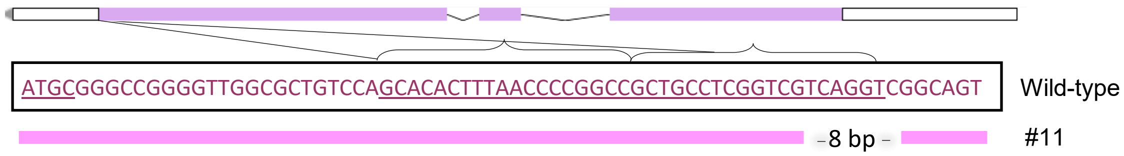

Supplementary Figure S2

A

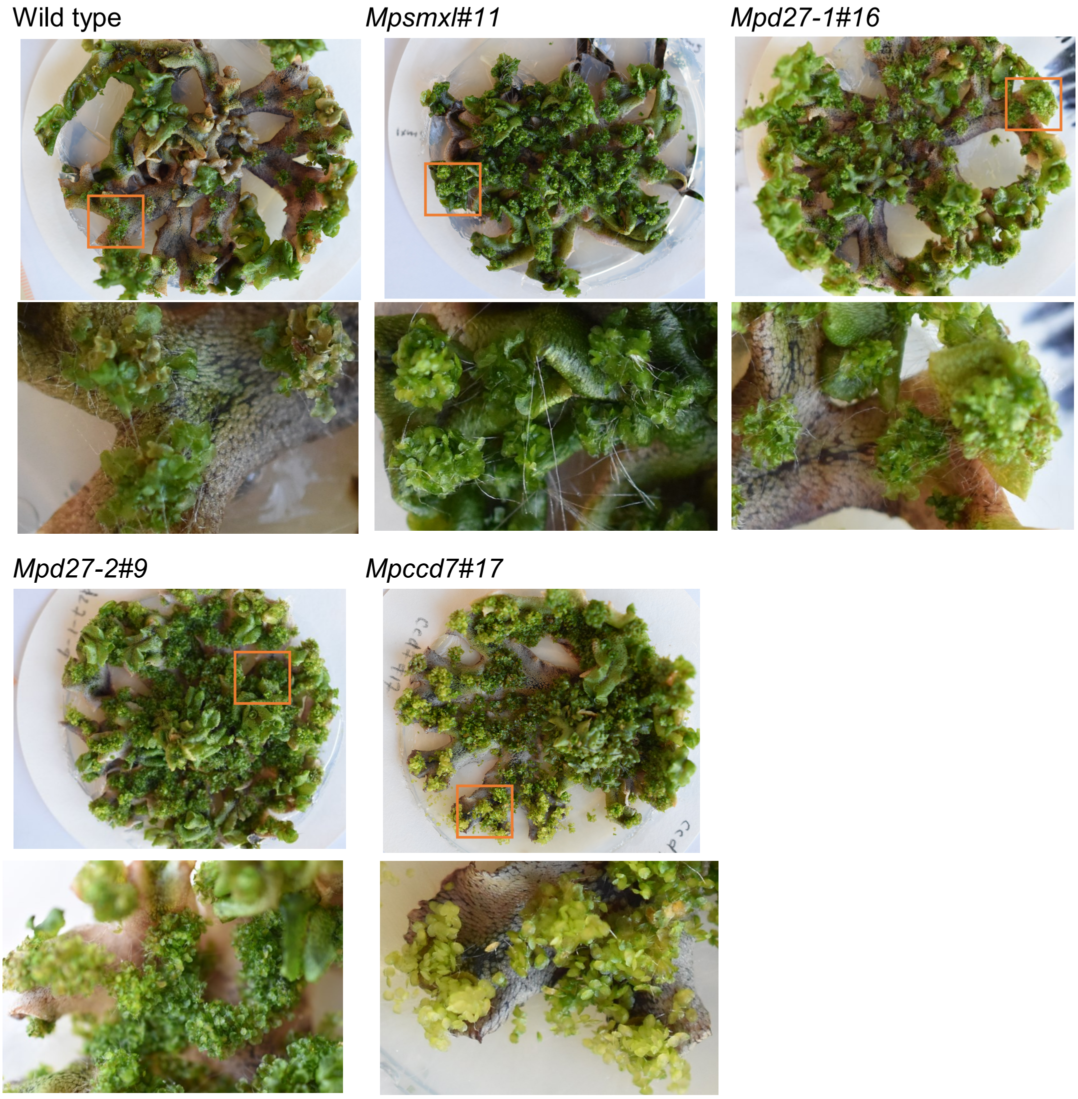

B

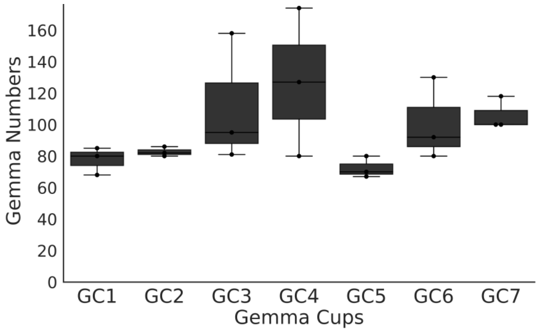

Supplementary Figure S3

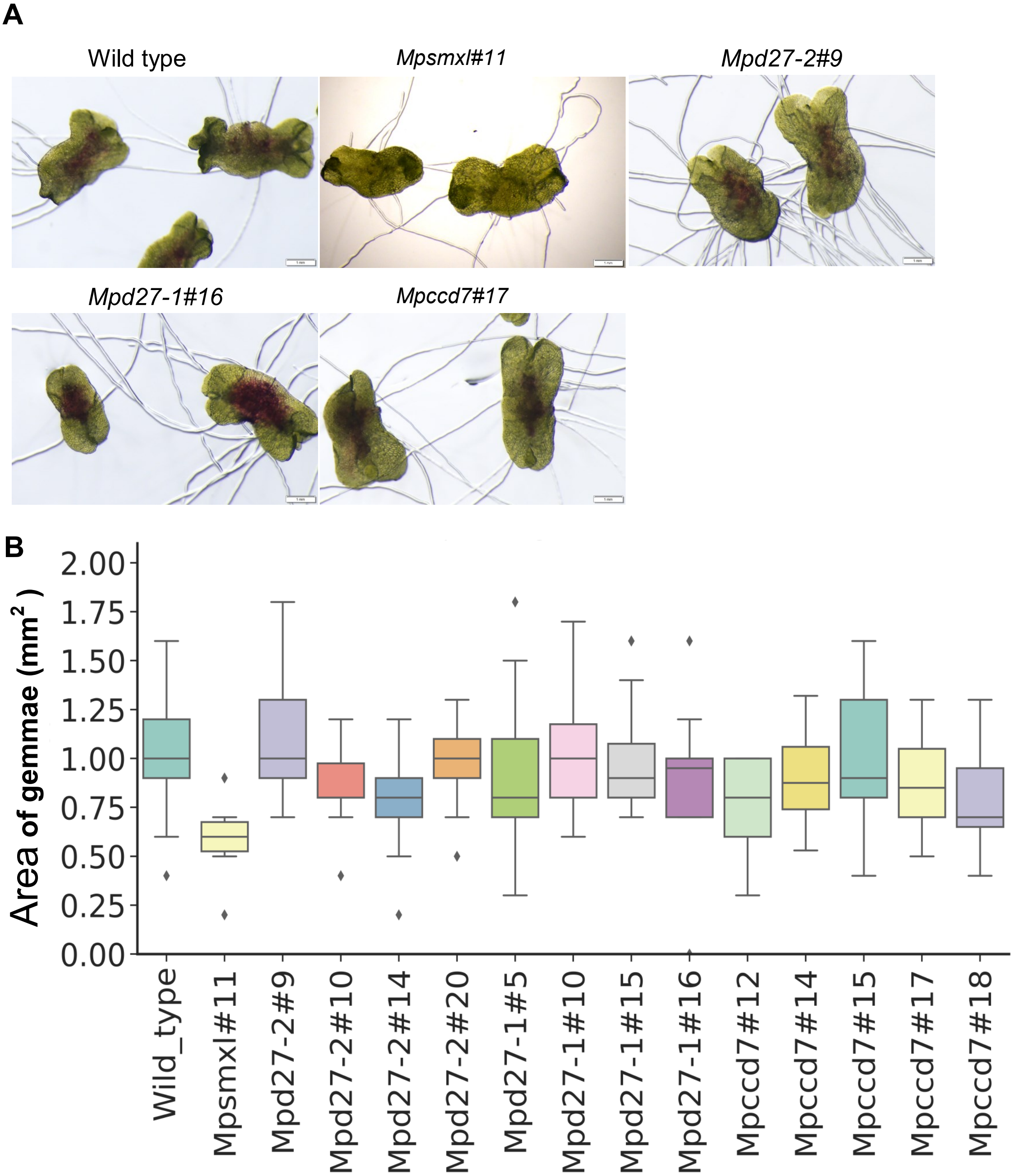

Supplementary Figure S4

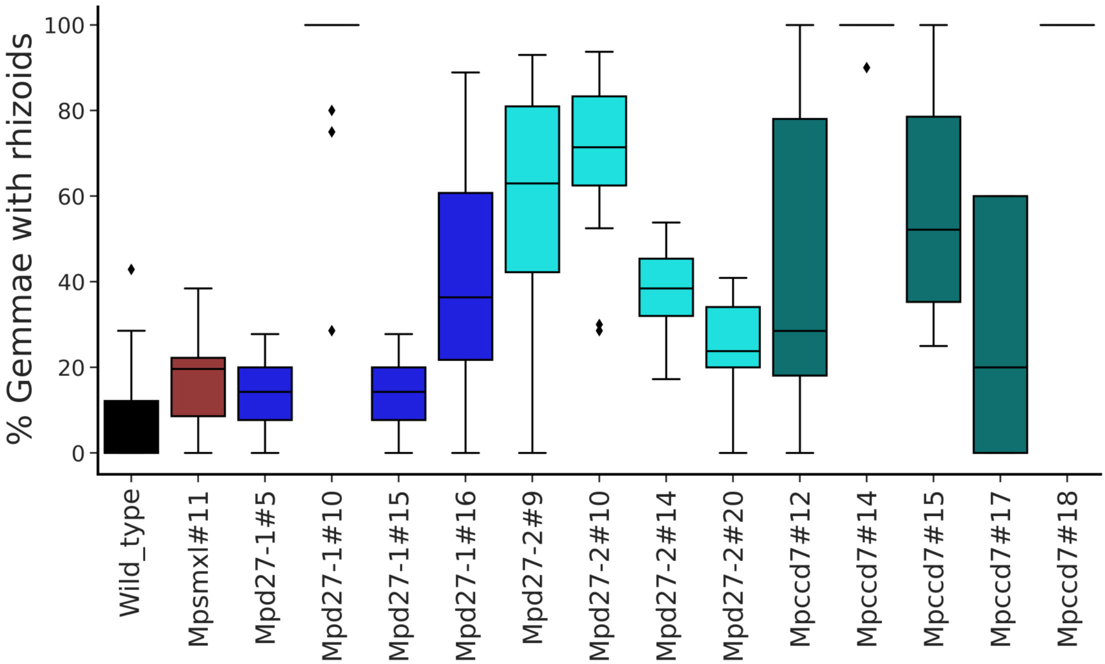

Supplementary Figure S5

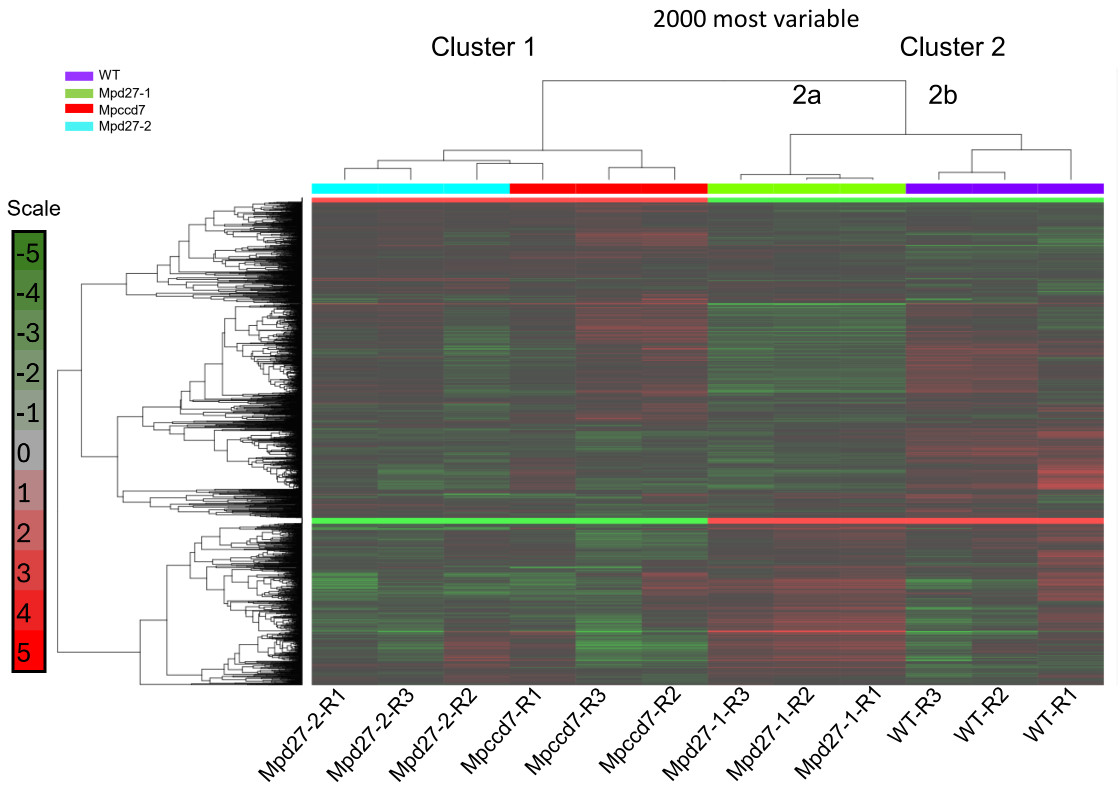

Supplement: Supplementary Figure 1 — CRISPR/Cas9-generated strigolactones mutants of Marchantia. DNA sequence of CRISPR-induced mutations in MpD27–1 (Mp6g03970), MpD27–2 (Mp6g01750), MpCCD7 (Mp2g03280) and MpSMXL (Mp3g06310). The underlined sequences enclosed by large black brackets represent the guides used, the symbol “—” represents the deletion, and the symbol “+ “represents insertions. The purple boxes represent exons, whereas the black lines represent the introns. [file Image_1.pdf]
